# The aging secretome as a candidate driver of bone remodeling imbalance: myokines, adipokines, and senescence-associated secretory factors

**DOI:** 10.3389/fendo.2026.1982491

**Published:** 2026-10-26

**Authors:** Hanhong Fang, Xianglong Zhou, Yang Liu, Weicheng Chen, Fulin Zhou, Haoran Zhou, Jianhui Xiang, Liming Xiong

**Affiliations:** Department of Orthopaedics, Union Hospital, Tongji Medical College, Huazhong University of Science and Technology, Wuhan, China

**Keywords:** bone remodeling, osteoblast–osteoclast coupling, aging secretome, myokines, adipokines, cellular senescence, senescence-associated secretory phenotype

## Abstract

Bone homeostasis depends on the spatial and temporal coupling of osteoclastic resorption to osteoblastic formation. With aging, this balance shifts toward net bone loss alongside sarcopenia, marrow adipose expansion, and chronic low-grade inflammation. These parallel changes may be linked by a shared set of secreted signals, termed here the aging secretome. We distinguish functional remodeling imbalance, defined as a sustained shift between formation and resorption, from direct uncoupling, in which a resorption-to-formation coupling signal is demonstrably disrupted. The aging secretome comprises three principal arms: muscle-derived signals, including myostatin, irisin, and myogenic extracellular vesicles; fat-derived signals, including leptin, adiponectin, and marrow adipocyte lipotoxicity; and senescence-associated secretory factors, particularly those involving senescent osteocytes, TNF-α, and IL-6. Inflammaging is treated as a shared contextual amplifier rather than a separate signaling arm. These pathways converge on key regulators of skeletal remodeling, including RANKL, Wnt–sclerostin, and IGF-1. This framework also provides a common context for comparing pathway-specific interventions, such as activin type II receptor blockade and sclerostin inhibition, with broader approaches targeting senescence or inflammation. An early phase 2 senolytic trial was negative overall, with only an exploratory signal in women with high senescent-cell burden. The aging secretome should therefore be viewed as an organizing framework whose individual pathways and relative contributions require further validation in human bone.

## 1 Introduction

Skeletal muscle, bone, and adipose tissue deteriorate together during aging, yet they are still largely studied as separate organ systems. Older adults who lose muscle also lose bone. The coexistence of osteoporosis and sarcopenia (1), termed osteosarcopenia, carries a higher risk of falls, fractures, and mortality than either condition alone (2, 3). Osteoporosis by itself already affects a large fraction of older adults, including nearly 30% of Chinese women aged 50 years and older (4), and it contributed to an estimated nine million osteoporotic fractures worldwide in the year 2000 (5). Two decades on the burden is larger: an estimated 178 million new fractures of all causes occurred worldwide in 2019, a 33.4% increase since 1990 (6), and disability-adjusted life-years attributable to low bone mineral density nearly doubled between 1990 and 2019, from 8.6 million to 16.6 million, with those from low-bone-mass-related fractures rising from 4.4 million to 9.8 million (7). Because these rises are driven mainly by population aging and growth, while age-standardized fracture rates fell by about a tenth over the same period (6), the clinical burden will continue to grow even where risk per head of population is stable (7). Concurrent muscle loss and adverse fat redistribution add to that burden. Excess or ectopic fat may join the decline as a third, metabolically active partner, and the number of older adults carrying these overlapping deficits continues to rise (8). The synchrony of these losses points to a possible common cause: shared signaling between the three tissues, as opposed to three independent aging trajectories.

Bone is a useful mechanistic anchor for examining that possibility. Adult bone mass is maintained by remodeling, in which osteoclastic resorption and osteoblastic formation are coupled in space and time, so that what is removed is replaced. Defined molecular signals enforce this coupling. TGF-β1 released from resorbed matrix recruits mesenchymal progenitors to remodeling sites (9), and matrix-derived IGF-1 then drives their differentiation into osteoblasts (10). Osteocytes govern the balance: they secrete receptor activator of nuclear factor-κB ligand (RANKL) to license osteoclastogenesis (11) and sclerostin to restrain Wnt-dependent formation (12). Aging tips this equilibrium toward net loss, as formation falls while resorption is sustained or increased. What drives that shift is unresolved.

Three streams of evidence, usually kept apart, bear on that question. Muscle secretes factors that reach bone. Myostatin, for example, suppresses osteoblast differentiation through osteocyte-derived exosomal miR-218 (13), and other myokines link contractile activity to bone formation (14). Adipose tissue is the second stream: leptin exerts central control over bone formation (15), while marrow adipose expansion diverts mesenchymal progenitors toward fat and exposes osteoblasts to lipotoxic fatty acids (16). Cellular senescence is the third. Aging bone accumulates senescent osteocytes with a pro-inflammatory secretome (17), and clearing these cells prevents age-related bone loss (18). Each stream has been characterized largely on its own, and few accounts treat the three as components of one signal.

Existing crosstalk reviews have generally considered these mediators according to their tissue of origin (19). Here, we use the term aging secretome to describe the proteins, extracellular vesicles, lipids, and metabolites released by aging muscle, adipose tissue, and senescent cells that have documented links to skeletal remodeling. These three sources were selected because they change with skeletal aging and signal to osteoblasts, osteoclasts, or osteocytes. Hepatic, intestinal, and broader osteokine inputs are outside the primary scope, as are most immune–endocrine pathways unless they directly contribute to inflammaging or senescence-associated crosstalk, as in the T-cell-derived TNF-α axis. The three sources therefore provide distinct routes to a common skeletal target, the remodeling unit. Their effects on formation and resorption are considered in the context of inflammaging, which may modify several of these signals simultaneously. This interpretation remains a working model rather than an established mechanism, although the reversal of age-related bone loss after senescent-cell clearance provides experimental support for a contribution of secretome-dependent signaling (18).

The present review is organized around the bone remodeling unit rather than around muscle, adipose tissue, and bone as parallel organs. This makes it possible to compare how muscle-derived, fat-derived, and senescence-associated signals reach osteoblasts, osteoclasts, and osteocytes, while distinguishing direct evidence on remodeling pathways from studies that report only net changes in bone mass or density. Senescence-associated secretory factors are considered alongside the muscle and fat compartments, whereas inflammaging is treated as a shared context that may modify signaling across all three. The same distinction is carried into the therapeutic section, where interventions are considered according to whether they act on a defined pathway or on broader inflammatory and senescence-related processes. In this respect, the review complements recent accounts that catalog bone–muscle mediators as parallel systems by making the source (20), skeletal target, direction of effect, and strength of evidence explicit for each relationship.

Sections 2–5 review the remodeling machinery and the evidence for muscle-, fat-, and senescence-associated signals. Section 6 considers how these findings can be integrated and where the proposed relationships remain uncertain, and Section 7 examines their therapeutic implications. Particular attention is given to conflicting evidence for mediators such as irisin and to the limited causal evidence currently available in humans.

### 1.1 Review scope and evidence classification

This article is a narrative review, not a systematic review, and it is intended to build a mechanistic framework rather than to pool effect sizes. We searched PubMed and Web of Science for English-language reports. The original search was run through 20 July 2026. Targeted update searches were then performed during revision, for topics raised in review and for newly published studies, through 17 September 2026. We combined terms for the remodeling unit (osteoblast, osteoclast, osteocyte, RANKL, sclerostin, coupling) with terms for the three arms (myokine, myostatin, irisin; adipokine, leptin, adiponectin, marrow adipose; cellular senescence, SASP, inflammaging) and for intervention (senolytic, sclerostin antibody, activin receptor, exercise). We placed no lower limit on publication year, tracing foundational work back through the reference lists of the retrieved reports. We prioritized primary experimental studies for mechanistic claims, human bone and clinical data, and pivotal randomized trials, and we used reviews only for orientation and for the mediator catalogs against which this synthesis is contrasted. Studies were excluded where the reported effect could not be matched to a bone outcome or to a defined tissue of origin. We did not register a reproducible, exhaustive search string, so this section defines a scope rather than a protocol. Where reports conflicted, as they do for irisin, adiponectin, and GDF11, we retained the disagreement and its likely sources instead of selecting a single direction.

To keep the strength of each claim visible, we grade the evidence behind every network edge on three axes. The first records where an effect was demonstrated: on a cell of the remodeling unit, on one arm without a demonstrated coupling link, or only as a net change in bone mass, density, or modeling. The second records the type of evidence available, from cell culture, to animal genetics or pharmacology, to human observational data, to human intervention; because these categories report different things, a human observational association is not treated as stronger mechanistic evidence than an animal genetic experiment. The third records the aging context, separating naturally aged animals and older or postmenopausal people from aging-adjacent models such as ovariectomy, hindlimb unloading, high-fat feeding, or focal irradiation, from disease settings, and from systems that are not aging-specific. Where an edge rests only on the latter three, its age-related behavior is presented as a candidate mechanism, and Sections 3, 4, and 5 each close by stating which links in that arm carry direct evidence from aged systems. **Table 1** applies this scheme edge by edge, recording direct cellular action, coupling evidence, the effect level, the aging context, and the type of evidence. Coding the first axis as an explicit column, rather than flagging it in prose, keeps well-supported mechanistic edges readable apart from associations that remain provisional. **Supplementary Table S1** then gives, for every study behind **Tables 1**, **2**, the study model, the population or experimental system with its sample size and sex composition where reported, the skeletal endpoint assayed, the principal limitation on interpretation, and the aging context. Read across the table, almost every secretome edge assembled here rests on single-arm evidence, which is why this review speaks of a secretome–remodeling network and reserves direct uncoupling for the narrower case defined in Section 2.

**Table 1:** Candidate mediators and contextual modifiers of remodeling imbalance and coupling: sources, target cells, separate effects on formation and resorption, and the evidence bearing on remodeling imbalance and coupling.

Factor (source)	Target cell	Effect on formation	Effect on resorption	Direct cellular action?	Coupling evidence?	Effect level	Evidence type/translational status	Aging context	Ref
Myostatin (muscle)	Osteocyte → osteoblast	↓	0	Yes (osteocyte→OB)	No	Single arm	Cell	Not aging-specific (cell lines)	(13)
GDF11 (muscle-associated)	Osteoblast/osteoclast	↑/↓ (unclear)	↑ (some)	Unclear	No	Both arms	Cell/animal	Natural aging (aged mice); aging-adjacent (OVX); not aging-specific (germline null)	(52–54)
Irisin (muscle)	Osteocyte/osteoclast	↑	↑ (context)	Yes (osteocyte/OC)	No	Both arms	Cell/animal/human obs.	Not aging-specific (young and transgenic mice, cell); aging-adjacent (OVX); natural aging (human obs.)	(14, 59, 61, 62)
IGF-1 (matrix-derived)	Osteoblast/progenitor	↑	0	Yes	Yes (matrix coupling factor)	Coupling	Animal/human obs.	Natural aging (aged rats; older human marrow); aging-adjacent (unloading and reloading)	(10, 64)
FGF2 (muscle-associated)	Osteoblast	↑	0	Yes	No	Single arm	Animal	Not aging-specific (germline null)	(65)
BAIBA (muscle)	Osteocyte	↑ (survival)	0	Yes (osteocyte)	No	Single arm	Cell/animal	Aging-adjacent (hindlimb unloading); natural aging (response lost with age)	(66)
EV miR-27a-3p (muscle)	Osteoblast progenitor	↑	0	Yes	No	Single arm	Cell	Not aging-specific (cell lines)	(67)
EV miR-196a-5p (muscle)	Osteoclast precursor/osteoblastic cell	↑	↓	Yes	No	Both arms	Cell	Not aging-specific (cell lines)	(70, 71)
IL-6 (inflammatory)	Osteoblastic cell (IL-6R)	0	↑ (indirect)	Yes (on osteoblastic cells, not OC progenitors)	No	Single arm	Cell/animal	Not aging-specific (co-culture, IL-6R transgenic)	(72)
IL-7 (inflammatory)	Osteoclast	0	↑ (context)	Yes (context)	No	Single arm	Cell/human obs.	Disease-associated (patients with solid tumors)	(73)
Leptin (fat)	Osteoblast (central/SNS)	↓	0	No (central/SNS)	No	Single arm	Animal	Not aging-specific (leptin-deficient and obese mice); aging-adjacent (OVX)	(15, 81)
Adiponectin (fat/marrow)	Osteoblast/osteoclast	↑/↓ (unclear)	↓/↑ (unclear)	Yes (*in vitro*)	No	Both arms	Cell/animal	Not aging-specific (knockout, cell); natural aging (young versus aged mice)	(82, 83, 102)
Resistin (fat)	Osteoclast	0	↑	Yes	No	Single arm	Cell	Not aging-specific (cell lines)	(85)
Free fatty acids (marrow adipocyte)	Osteoblast	↓	0	Yes	No	Single arm	Cell/animal/human obs.	Not aging-specific (human co-culture); disease-associated and aging-adjacent (type 2 diabetes, high-fat feeding)	(99, 101)
RANKL (marrow adipocyte)	Osteoclast	0	↑	Yes (resorption arm)	No	Single arm	Cell (human)	Not aging-specific (glucocorticoid-driven human cells)	(90)
LepR^+^ MSC fate switch (context)	Progenitor	↓	0	Context (cell fate)	No	Single arm	Animal	Not aging-specific (conditional genetics, adult fate mapping); aging-adjacent (high-fat diet, irradiation)	(86, 87)
SASP: IL-6, TNF-α (senescent cells)	Osteoblast + osteoclast	↓	↑	Yes (both arms)	No	Both arms	Animal	Natural aging (old versus young mice; aged mice with established bone loss)	(17, 18)
T-cell TNF-α, estrogen loss (immune)	Osteoclast (via RANKL)	0	↑	Yes (via RANKL)	No	Single arm	Animal	Aging-adjacent (ovariectomy throughout)	(135, 137, 138)
TNF-α background modifier: Runx2↓, Dkk-1↑ (inflammatory)	Osteoblast/myokine edge	↓	↑ (indirect)	Modifier (edge inversion)	No	Both arms	Cell/animal/human obs.	Not aging-specific (calvarial and cell lines); disease-associated (inflammatory arthritis)	(154–157)
Osteocalcin (bone→muscle, reverse)	Myofiber	—	—	Reverse edge	—	Reverse edge	Animal/human obs.	Natural aging (age-related decline; aged mice restored)	(153)

Edge list of the secretome–remodeling network shown in **Figure 3**. Each row is one factor (one network edge), giving its source, principal target cell, and separate effects on formation and on resorption (↑ increase, ↓ decrease, 0 no clear effect, unclear when reported signs conflict). ‘Direct cellular action?’ records whether the factor acts directly on a cell of the remodeling unit (Yes, No, Unclear; Context for a cell-fate step; Reverse for a bone-to-muscle edge), and ‘Coupling evidence?’ is marked Yes only where a resorption-to-formation coupling signal is itself altered, so a factor acting on both arms is not counted as direct coupling evidence. ‘Effect level’ states the level at which each edge is evidenced: Coupling where a coupling signal itself is altered, Single arm where the demonstrated effect is confined to formation or to resorption, Both arms where independent effects on each arm are reported without a demonstrated coupling link, and Reverse edge for the bone-to-muscle entry. Read down this column, almost every secretome edge is evidenced at the single-arm or both-arms level and none at the coupling level, which is why the framework is described as a secretome–remodeling network and direct uncoupling is reserved for the narrower case defined in Section 2. ‘Evidence type/translational status’ lists every evidence type available for that edge (Cell, Animal, Human obs., Human interv.), taken as the union of the study models recorded for its cited studies in **Supplementary Table S1** rather than as a single principal design; these categories are not ranked (see Section 1.1). ‘Aging context’ records the aging status of the systems in which each edge was demonstrated: Natural aging for aged animals or for older or postmenopausal people studied without an intervening manipulation, Aging-adjacent for models that reproduce features of aging (ovariectomy, hindlimb unloading, high-fat feeding, focal irradiation), Disease-associated for evidence obtained in a disease setting (type 2 diabetes, inflammatory arthritis, malignancy), and Not aging-specific for cultured cells, young or transgenic animals, and germline or conditional genetic models; a row carries more than one value where the cited studies differ. Counted this way, 7 of the 20 edges have any evidence from naturally aged systems and only 2 rest on such evidence alone, the senescent-cell SASP row and the reverse osteocalcin edge, while 8 depend in part on aging-adjacent models and 14 include evidence that is not aging-specific. The age-related behavior of the remaining edges is accordingly presented in Sections 3 to 5 as a candidate mechanism. Most factors are external secretome inputs, whereas IGF-1 is a local matrix coupling factor and osteocalcin is a reverse bone-to-muscle edge. Signs are further modified by sex, menopausal stage, skeletal site, dose, and turnover state, as discussed in Section 6.2. Numbers in parentheses refer to the numbered reference list; abbreviations are defined in the Abbreviations section.

**Table 2:** Human evidence linking the three secretome arms to bone outcomes.

Arm	Marker/factor	Population/sex	Design	Bone outcome	Direction/key finding	Ref
Muscle	Serum myostatin	Healthy postmenopausal women, n = 175, 51–75 y (F)	Cross-sectional	BMD, body composition	No difference by osteoporosis status after age adjustment; no correlation with BMD (null result)	(77)
Muscle	Serum myostatin/irisin	Rheumatoid arthritis, n = 182 vs 142 controls; sex NR	Cross-sectional	Sarcopenia; osteoporotic fracture	Myostatin ↑ and irisin ↓ in the sarcopenia and in the fracture subgroups, each versus its own comparator	(78)
Muscle	Serum irisin	Overweight postmenopausal women, n = 36 vs 36 (F)	Cross-sectional	Prevalent vertebral osteoporotic fracture	Low irisin linked to fracture, independent of BMD	(62)
Fat	Marrow adipose tissue	Adults 30–100 y, n = 53, plus 26 with osteoporosis; sex NR	Iliac-crest biopsy, histomorphometry	Marrow fat volume fraction	Rises with age and further in osteoporosis	(79)
Fat	Vertebral marrow fat	Older men, n = 82, 67–101 y (M); and patients n = 51, 28 F (mixed)	MRS; PDFF imaging	Vertebral marrow fat	Higher in osteoporosis	(103, 104)
Fat	Vertebral marrow fat	Patients n = 52, 63–70% women (mixed)	PDFF imaging, fracture case-control	Osteoporotic vertebral fracture	Fat fraction separates fracture from non-fracture	(107)
Fat	Serum leptin	Healthy adults, 45 studies pooled (mixed)	Meta-analysis	BMD, BMC	Positively associated with BMD, most clearly in postmenopausal women	(110)
Fat	Serum adiponectin	Community-dwelling postmenopausal women, n = 386 (F)	Cross-sectional	BMD	Inversely related to femoral-neck BMD in women with osteoporosis, adjusted for body composition	(112)
Fat	Serum adiponectin	Older men, n = 989, MrOS Sweden (M)	Prospective, mean 5.3 y	Incident osteoporotic fracture	High adiponectin predicts fracture; gradient of risk wanes with time since baseline	(114)
Senescence	p16/p21/SASP markers	Elderly postmenopausal women, n = 10 on estrogen therapy vs 10 untreated (F)	Bone biopsy, estrogen-therapy comparison	Senescence biomarkers, SASP	Unchanged by estrogen therapy, placing senescence independent of sex-steroid status; no young comparator	(127)
Senescence	Inflammatory bone proteome	Children, middle-aged, and elderly donors; sex NR	Bone proteomics across age groups	Tissue signaling	Inflammatory pathways up, formation signals down with age	(129)
Senescence	Marrow RANKL^+^ cells	12 premenopausal, 12 early postmenopausal, 12 estrogen-treated postmenopausal women (F)	Cross-sectional, flow cytometry	Surface RANKL per marrow cell	Two- to threefold higher after menopause; correlates with resorption markers	(136)

Human evidence for the three arms of the aging secretome, consolidated from Sections 3–5 and summarized in Section 6.2. Each row gives one human marker or factor, the study population and design, the bone outcome measured, the reported direction of association, and supporting references. ‘Population/sex’ records the cohort with its sample size and its sex composition as reported in the source, marked F, M, or mixed, and marked NR where the source itself does not report the composition; ‘Design’ records the study design separately, so that the population and sex dimension can be read independently of design. Sex-dependent effects are discussed in Section 5.4. The table collects the human data underlying the mechanistic edges of **Figure 3** and **Table 1**. Directions are reported as published, including the null result for serum myostatin, and the entries are observational except for the estrogen-therapy comparison in the senescence arm; none of them establishes causality, which for that arm rests on the reversal experiments discussed in the text. Numbers in parentheses refer to the numbered reference list. BMD, bone mineral density; MRS, magnetic resonance spectroscopy; PDFF, proton-density fat fraction; RANKL, receptor activator of nuclear factor-κB ligand; SASP, senescence-associated secretory phenotype.

Two conventions govern how the three arms are delimited, and they are stated here because they determine how Sections 3 to 5 should be read. The first concerns molecules that arise from more than one source. Each edge is defined by the combination of its source, temporal pattern, and receptor route, so the same molecule can represent distinct edges when these features differ and a skeletal relationship is separately evidenced. Interleukin-6 is considered in three biological contexts: acute release from contracting muscle, sustained production during chronic tissue inflammation, and production as part of the senescence-associated secretory phenotype. The acute muscle context is retained as a contextual contrast, whereas **Table 1** lists the source-defined skeletal IL-6 relationships used as network edges. On the same convention, inflammaging is not a fourth arm and not a target node, but a global contextual modifier that may alter the strength or direction of several source-defined edges at once, a position developed in Section 6.1. Treating it this way also prevents the cytokines shared between the arms from being counted twice.

The second convention concerns senescence, which is not confined to any one tissue. Senescent cells accumulate in muscle, adipose tissue, bone, and the immune compartment alike. The senescence arm as used here denotes the secretory phenotype of senescent cells accumulating within bone and marrow, principally osteocytes, osteoprogenitors, and marrow immune cells. Senescence arising in muscle or in adipose tissue is not assigned to this arm; it is treated as part of the age-dependent change in the muscle and fat arms themselves, and is discussed in Sections 3.5 and 4.5. The distinction is one of source rather than of mechanism, since the mediators involved overlap substantially, and it is adopted so that each edge has a single defined tissue of origin.

## 2 The coupling paradigm and its imbalance in aging

Adult bone mass is continuously renewed through remodeling, and the defining feature of that process is the coupling of resorption to formation. In each cycle a basic multicellular unit of osteoclasts excavates a packet of mineralized matrix, and osteoblasts refill it, so that under healthy conditions the amount removed is matched by the amount replaced. Three cell types set the terms of this exchange: osteoclasts of hematopoietic origin, osteoblasts derived from mesenchymal progenitors, and osteocytes. Osteocytes are terminally differentiated osteoblasts embedded in the matrix, and they act as the principal coordinators of the unit. They are now recognized as the dominant source of the RANKL that permits osteoclast formation (11, 21), which places the control of resorption inside the same cell network that directs formation. Coupling is therefore a single regulated system whose output is net bone mass, and not two independent programs that happen to balance (22, 23).

Coupling is enforced by a defined set of molecular signals that link the phases of remodeling. Resorption itself supplies part of the formation signal: transforming growth factor-β1 released from degraded matrix recruits mesenchymal progenitors to the resorption site (9), and insulin-like growth factor-1 from the same matrix then drives their differentiation into matrix-producing osteoblasts (10). The magnitude of resorption is set by the balance between RANKL and its decoy receptor osteoprotegerin (OPG). Osteoprotegerin was identified as a secreted TNF-receptor-family protein that restrains resorption and raises bone density (24). Its ligand RANKL is the cytokine that drives osteoclast differentiation and activation (25, 26). Genetic deletion of RANKL produces severe osteopetrosis with a complete absence of osteoclasts, which establishes the axis *in vivo* (27, 28). The link also runs in the reverse direction. Vesicular RANK shed by maturing osteoclasts binds RANKL on osteoblastic cells and triggers a reverse signal that activates Runx2 to promote formation (29), so the two arms are reciprocally connected and not merely sequential. Formation is held in check by the Wnt antagonist sclerostin, which osteocytes secrete to restrain osteoblast activity and which mechanical loading suppresses to release that brake (12). Human LRP5 mutations show how much Wnt signaling matters here. These lower or raise pathway activity and produce mirror-image low- and high-bone-mass phenotypes (30, 31), establishing canonical Wnt signaling as a central regulator of bone mass (32). Osteoblast-derived WNT16 illustrates the specificity of this control: it represses osteoclastogenesis and protects cortical bone from fragility fractures (33). These signals are the adjustable set points of the coupled unit, and upstream endocrine and paracrine inputs can move them.

Aging leaves this machinery intact but biases the balance toward loss. The osteogenic capacity of the mesenchymal lineage declines with age, while the same progenitors are increasingly diverted toward adipocytes, so marrow fills with fat as bone-forming potential falls (16). A proximal driver is the age-associated rise in reactive oxygen species, which impairs the generation and survival of osteoblasts and osteocytes and has been advanced as a mechanism of skeletal aging more fundamental than the loss of sex steroids (34, 35). On the resorption side, osteocyte-derived RANKL is sustained or elevated in the aging skeleton, so osteoclastogenesis stays licensed even as formation weakens. Osteoclasts from aged bone also begin to express sclerostin, which adds a further brake on formation (36). Reduced physical activity compounds the change, since the loading stimulus that normally suppresses sclerostin and drives formation diminishes; the sclerostin response to unloading shows how quickly formation can be turned down when mechanical input is withdrawn (37). Superimposed on these changes is the accumulation of senescent cells within bone, a persistent local source of pro-inflammatory and pro-resorptive signals (17). The net result is a remodeling balance that runs negative, with resorption sustained and formation diminished, which is the functional readout of an imbalanced unit. We adopt a two-level terminology to keep the mechanism precise. We use functional remodeling imbalance for a net negative balance that arises when formation is independently suppressed, resorption is independently enhanced, or bone mass is otherwise driven negative. We reserve direct uncoupling for the narrower case in which the signals that link resorption to formation are themselves disrupted. We invoke it below only where such a disruption has been shown, and describe secretome effects on a single arm as functional remodeling imbalance otherwise. A further distinction is needed between remodeling and modeling. Remodeling is the coupled resorption-and-formation cycle within the basic multicellular unit, whereas modeling is the independent addition or removal of bone at separate surfaces, as in periosteal apposition. Some of the evidence discussed below reports periosteal modeling, cell survival, the response to mechanical loading, or net changes in bone mineral density, none of which by themselves establish an event inside the remodeling unit or a change in resorption-to-formation coupling. **Table 1** grades each edge on the three levels set out in Section 1.1. **Figure 1** places the homeostatic unit beside its aged, imbalanced counterpart.

**Figure 1:**
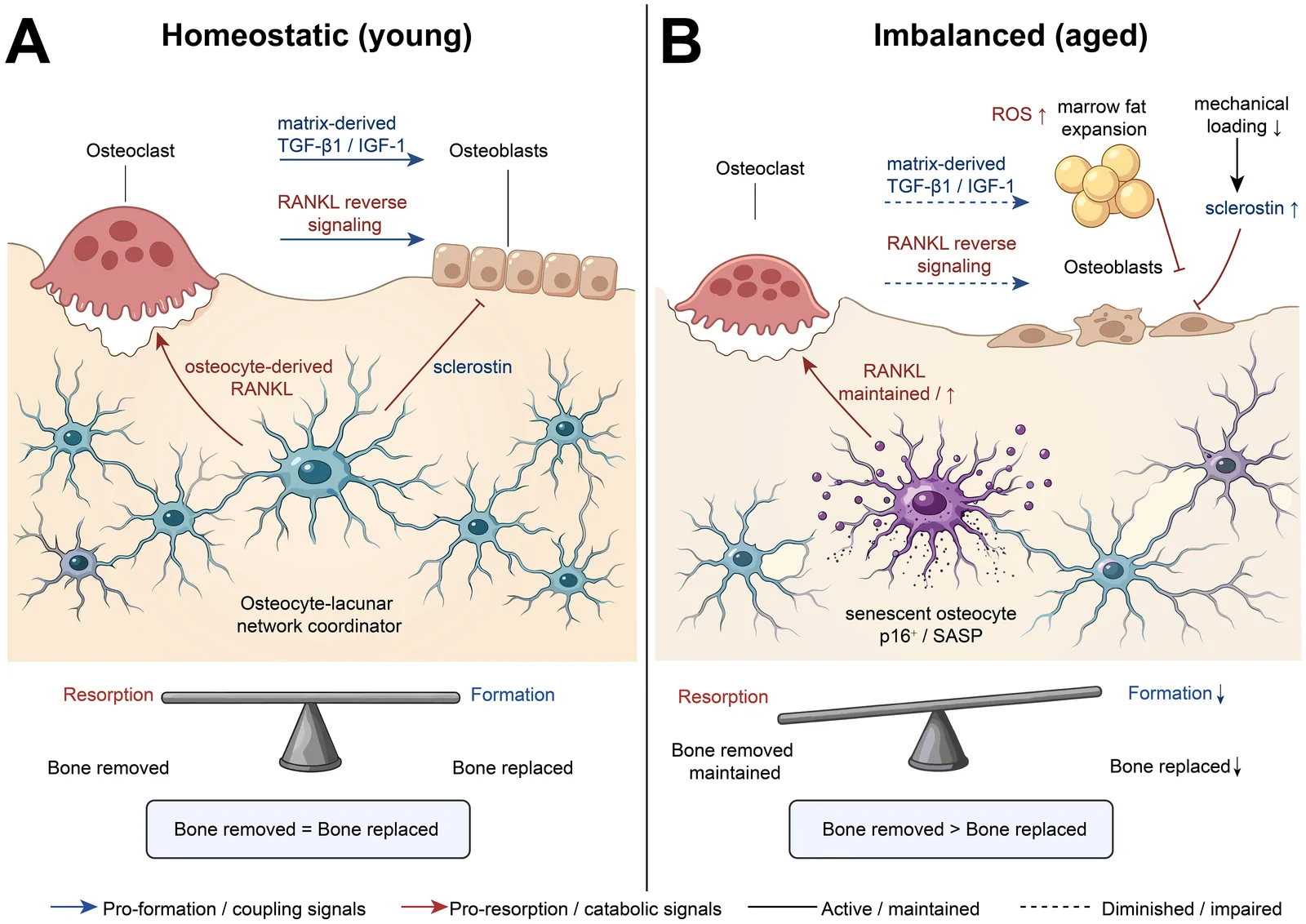
The coupled remodeling unit and its imbalance in aging. The bone multicellular unit couples resorption to formation so that the bone removed by osteoclasts is matched by the bone replaced by osteoblasts, with the osteocyte–lacunar network serving as the central coordinator and a balance beam denoting net bone mass. **(A)** In the homeostatic (young) unit, resorption and formation are balanced through matrix-derived TGF-β1 and IGF-1, osteocyte-derived RANKL and sclerostin, and RANKL reverse signaling, so that bone removed equals bone replaced and the balance remains level. **(B)** In the aged unit, rising reactive oxygen species, senescent osteocytes bearing a pro-inflammatory secretome, sustained or elevated RANKL, osteoclast-derived sclerostin, marrow adipose expansion, and reduced mechanical loading weaken the formation arm while resorption persists, tilting the balance toward net loss. This selective failure of formation with maintained resorption is the cellular signature of functional remodeling imbalance. Blue denotes pro-formation (anabolic) signals and red denotes pro-resorption (catabolic) signals; line style distinguishes active from diminished signals between the two states.

The coupled remodeling unit provides a common endpoint through which diverse upstream signals can influence bone mass. However, the temporal sequence of age-related changes remains uncertain, and it is not established whether reduced formation, increased resorption, or accumulation of senescent cells occurs first. This uncertainty limits identification of the most appropriate point for intervention. In addition, as summarized in **Table 1**, most secretome-related evidence concerns changes in formation or resorption rather than direct disruption of resorption-to-formation coupling. The following sections therefore focus on remodeling outcomes while tracing muscle-, fat-, and senescence-derived signals to their skeletal targets.

## 3 Muscle-derived and muscle-associated signals: the myokine arm

### 3.1 Myokines as systemic regulators of the remodeling unit

Skeletal muscle is an endocrine organ, and the factors it releases, collectively termed myokines, reach bone and influence its cells directly or indirectly (38–41). This signal travels both as free protein and within extracellular vesicles, as Section 3.4 details. These factors distribute across the whole skeleton and are not confined to the myotendinous interface, so muscle acts as a systemic modulator of bone turnover, consistent with the long-recognized coordinated aging of the bone–muscle unit (42–44). Not every factor discussed here is exclusively muscle-derived. Some, such as GDF11, FGF2, and the inflammatory cytokines, are muscle-associated or systemic signals whose bone effects are shared with other sources, and we flag this distinction where the muscle origin is not firmly established. The myokines mapped onto bone fall into two functional groups whose actions are opposite in sign: one suppresses osteoblastic formation and the other promotes it, while several inflammatory myokines act mainly on the resorptive side. **Figure 2A** maps these myokines onto the shared target cells and marks each by its net sign on formation or resorption. Grouping the muscle arm by net effect on formation versus resorption, instead of by molecular family, shows how a change in the muscle secretome can move the coupled unit in either direction. It also anticipates the central problem of aging, since healthy muscle and sarcopenic muscle present bone with different profiles. The factors mapped here are those with defined skeletal targets, and they do not exhaust the muscle secretome. A wider panel with reported but unevenly graded bone effects includes apelin, METRNL, SPARC, musclin, and GDF15; a recent evidence-based classification of exercise-induced myokines, graded by mechanistic support and translational readiness, places apelin among the better-supported mediators, METRNL among those with defined receptors but largely correlative human data, and SPARC among exploratory candidates (45), while GDF15 is higher in older women with osteoporosis than in those without, varies inversely with hip bone mineral density and with distal radius bone quality, and tracks muscle expression of TNF-α and IL-1β, which ties it to the inflammatory background rather than to a single skeletal direction (46). These mediators are not entered in **Table 1**, since the effect signs that table requires are precisely what remains undetermined for them.

**Figure 2:**
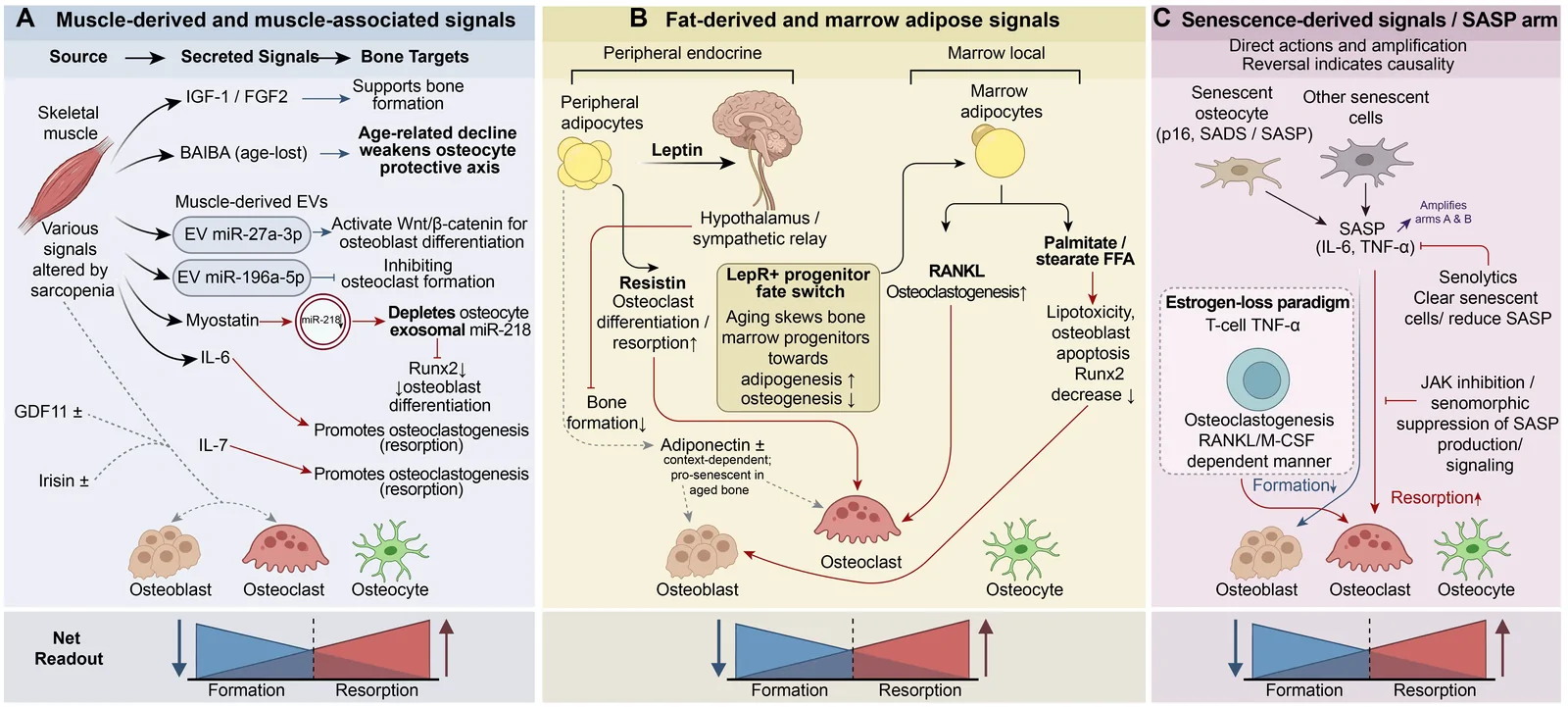
The three effector arms of the aging secretome and their net actions on the coupled unit. Muscle-derived and muscle-associated **(A)**, fat-derived **(B)**, and senescence-derived **(C)** signals converge on a shared set of target cells (osteoblast, osteoclast, and osteocyte), with the net formation-versus-resorption readout shown at the base of each panel. **(A)** Muscle arm: myostatin suppresses osteoblast differentiation through osteocyte exosomal miR-218 and Runx2, whereas GDF11 is shown as a context-dependent signal with conflicting reported effects; IGF-1, FGF2, BAIBA (age-lost), and the extracellular-vesicle microRNAs miR-27a-3p and miR-196a-5p support formation or restrain resorption; IL-6 and IL-7 promote resorption, and irisin acts in a context-dependent manner. **(B)** Fat arm: leptin suppresses formation through a central sympathetic relay, resistin and marrow-adipocyte RANKL promote resorption, and saturated free fatty acids are lipotoxic to osteoblasts; the LepR^+^ progenitor is diverted from bone toward fat, and adiponectin is context-dependent and turns pro-senescent in aged bone. **(C)** Senescence arm: senescent osteocytes secrete a senescence-associated secretory phenotype (IL-6, TNF-α) that lowers formation and raises resorption. The boxed estrogen-loss paradigm is shown separately, as an illustration in which T-cell TNF-α drives RANKL- and M-CSF-dependent osteoclastogenesis. Independently of that paradigm, senolytic clearance of senescent cells and JAK-inhibitor suppression of SASP production raise bone mass in aged mice, an effect that is agent-specific (Section 7.3). Blue arrows denote pro-formation and red arrows denote pro-resorption actions; gray dashed arrows mark context-dependent mediators (irisin, adiponectin, GDF11) whose sign varies with dose, target, and background. Because arm C contributes IL-6 and TNF-α to the shared inflammaging milieu, it remains a distinct senescence-derived arm while also amplifying signals from arms A and B (purple).

### 3.2 The suppressive arm: myostatin and the TGF-β superfamily

The TGF-β superfamily myokines, with myostatin as their prototype (47), act mainly to suppress the formation arm of remodeling. Myostatin does more than mirror muscle mass; it signals to bone directly. It reprograms osteocytes to release exosomes depleted of miR-218, and the resulting shortfall of miR-218 delivery to osteoblasts lowers Runx2 and blunts osteoblastic differentiation (13). Loss-of-function models show the skeletal weight of this pathway. Genetic deletion of myostatin raises bone mineral content and strength (48), an effect reproduced by combined pharmacological inhibition of myostatin and activin A in a mouse model of osteogenesis imperfecta (49), and the same deletion amplifies the bone-strength gains achieved through exercise (50). Endogenous antagonism reinforces the point: decorin neutralizes myostatin and upregulates follistatin, so the balance reflects agonists and inhibitors together, not ligand concentration alone (51). GDF11, a close myostatin paralog, shows how contested a single myokine can be. Some genetic evidence indicates that GDF11 promotes osteogenesis and that its inhibitor follistatin builds muscle at the expense of bone (52). Other work reports the opposite: that GDF11 decreases bone mass by stimulating osteoclastogenesis and suppressing osteoblast differentiation (53, 54). This disagreement, traceable in part to the near-identical biochemistry of recombinant GDF11 and myostatin, warns against assigning a single sign to a myokine. The dominant reading of this arm nonetheless is a suppressive pressure on formation: when muscle raises its output of myostatin-family ligands, the formation side of the coupled unit yields first.

### 3.3 The anabolic arm: irisin, IGF-1, and metabolite signals

A second group of myokines promotes bone formation. Its best-studied member, irisin, shows how unsettled the net effect of a myokine can be. Irisin is cleaved from FNDC5 during exercise (55). Given to mice, it increases cortical bone mass (14, 56, 57) and both prevents and restores disuse-induced bone loss in hindlimb-suspended animals (58). It acts through αV integrin receptors on osteocytes, and here the picture turns. Deleting FNDC5 blocks the osteocytic osteolysis and bone loss that follow ovariectomy, which implies that irisin can also permit resorption in that setting (59), and it protects female mice from the same two processes under lactation or dietary calcium restriction (60). The tension becomes explicit in direct evidence that irisin stimulates osteoclastogenesis and bone resorption *in vitro* and *in vivo*, an action blocked by neutralizing αVβ5 integrin (61). In humans, low circulating irisin has been associated with prevalent osteoporotic fractures independently of bone mineral density, although its relationship with density itself is inconsistent (62). Whether irisin is a net anabolic or catabolic signal therefore depends on dose, target cell, and setting, and this conflict is among the clearest open questions in the muscle–bone field (63). Other anabolic inputs are less ambiguous. IGF-1 reaches bone from muscle, from resorbed matrix, and from the circulation, and in mature osteoblasts it is required for the periosteal bone formation that follows mechanical reloading (64). FGF2 supports bone mass and formation, since its deletion reduces both (65); both are muscle-associated signals rather than strictly muscle-derived ones. The muscle metabolite β-aminoisobutyric acid protects osteocytes from reactive-oxygen-species-induced death, and this protection is itself lost with age through reduced receptor expression, so an anabolic muscle signal fails in the very setting where it is needed (66). The anabolic arm is therefore a counterweight to myostatin-family suppression, but a conditional one, and in the case of irisin it may act on both sides of the coupled unit at once.

### 3.4 Vesicular transport and inflammatory myokines

A large part of the muscle-to-bone signal travels inside extracellular vesicles rather than as free protein, and this cargo links the muscle arm to the inflammatory signals taken up in Section 5. Muscle-derived vesicles enriched in miR-27a-3p are taken up by osteoblast progenitors, where they activate Wnt/β-catenin signaling and promote osteogenic differentiation (67, 68). Loss of muscle input reshapes this output, since sciatic denervation alters muscle FNDC5 and FGF21 expression and accompanies bone loss (69). Vesicles from muscle cells can also suppress osteoclast formation by acting on the mitochondrial metabolism of osteoclast precursors (70), an effect attributable in part to their miR-196a-5p content, which in the same work enhanced osteoblastic differentiation in stromal and pre-osteoblastic lines, so this cargo acts on both arms at once (71). The direction of the vesicle signal is therefore itself conditional, consistent with the mixed picture seen for individual myokines.

Overlaid on this transport system are the inflammatory myokines, which act mainly to raise resorption. Interleukin-6 shows why source and time course matter. The acute, pulsatile IL-6 released by contracting muscle is not equivalent to the chronic IL-6 of inflammaging; the two differ in receptor route and in net skeletal effect. The resorptive action described here, mediated through IL-6 receptors displayed on osteoblastic cells rather than on osteoclast progenitors, refers to the sustained inflammatory pool (72). By the convention set out in Section 1.1, the sustained inflammatory pool and the senescence-derived pool of the same cytokine are treated as separate source-defined edges rather than as one molecule with an averaged effect, the latter in Section 5.3, while the acute muscle context is retained as a contextual contrast rather than tabulated as an edge of its own. Interleukin-7 promotes osteoclastogenesis in a TNF-α-dependent manner (73), although the evidence that skeletal muscle is a quantitatively important source is limited, and its net skeletal effect is likely context-dependent. Because these mediators are also part of the aging inflammatory environment, the muscle arm and the senescence arm share signaling molecules and converge on the same coupled unit.

### 3.5 The muscle arm across the aging trajectory

The clinical significance of the muscle arm emerges when its two sides are read against the trajectory of aging. Sarcopenic muscle shifts its secretory profile toward the catabolic pole: myostatin tends to rise while exercise-dependent anabolic myokines such as irisin tend to fall. Muscle loss thus converts a tissue that normally supports bone into one that adds to its decline. This reciprocity gives a mechanistic basis for the clinical coupling of sarcopenia and osteoporosis, the combined entity of osteosarcopenia, which affects roughly one in six postmenopausal women without a demonstrated rise in prevalence across study-level mean age (74), carries elevated fracture and mortality risk (75), and predicts incident frailty (76). Human data are more equivocal than the animal work, and they do not simply echo this shift. In 175 healthy Chinese postmenopausal women, serum myostatin did not differ between those with and those without osteoporosis once age was taken into account, and it correlated with neither body composition nor bone mineral density at any site (77). A disease cohort reads differently. Among patients with rheumatoid arthritis, myostatin was higher and irisin lower in those with sarcopenia than in those without, and the same direction appeared in those with osteoporotic fracture compared with those without (78). The irisin side stays less settled, because low circulating irisin has been linked to prevalent fracture independently of density yet shows no consistent relation to density itself. Circulating myokines therefore do not yet reproduce in healthy aging humans the suppressive pressure on formation that the animal work describes, and the clearest human signals come from a disease cohort and from fracture rather than from density.

The human data also identify the myostatin–activin axis and the vesicle transport system as points where the muscle-to-bone signal might be redirected, a possibility taken up in Section 7. Two gaps constrain that prospect. First, the net skeletal effect of irisin must be resolved before it can be treated as a target rather than a confound. Second, the specificity of vesicle-based delivery remains poorly defined, meaning how muscle vesicles are addressed to bone and not to other organs, which bears on whether the pathway can be manipulated without off-target effects.

Two kinds of evidence support this arm, and they should not be read as one. Part of it comes from naturally aged tissue: GDF11 lowers bone mass in aged as well as young mice (53, 54), matrix IGF-1 falls in the bone of aged rats and in the marrow of older adults with osteoporosis (10), and the skeletal response to BAIBA is lost with age (66). The remainder rests on cultured cells, young or transgenic mice, and unloading or ovariectomy models, while the human myokine data are cross-sectional and, for myostatin in healthy postmenopausal women, null. Those links enter the framework as candidate mechanisms for the aging skeleton, and **Table 1** records the aging context of each edge. Adipose tissue raises related questions, and its expansion within the marrow introduces a second, overlapping set of secreted signals acting on the same coupled unit.

## 4 Fat-derived signals: the adipokine and marrow adipose arm

### 4.1 Two routes from fat to bone

Adipose tissue influences bone through two distinct routes that a single organ label obscures. As an endocrine organ, body fat releases adipokines that circulate to bone and act on the remodeling unit. As a local depot, marrow adipose tissue occupies the same niche as bone-forming progenitors and communicates with them at short range. The clinical picture is counterintuitive, since more fat does not mean stronger bone. Marrow fat expands precisely when bone is lost, rising with age and further in osteoporosis in human iliac-crest biopsies (79, 80). Obesity-associated changes in bone quality can also accompany a bone mineral density that appears preserved, so quality and location describe the fat–bone relationship better than mass does. In the aging body both routes tend to bias the coupled unit toward resorption and away from formation, and the sections below follow the circulating adipokines and then the marrow depot. **Figure 2B** sets out the circulating adipokines and the marrow depot on the same target cells, and marks the net sign each carries.

### 4.2 Circulating adipokines and their conflicting signs

Circulating adipokines act on both arms of remodeling, and their reported signs are often in conflict, an ambiguity that parallels the irisin problem of the muscle arm. Leptin exerts a powerful central effect. Acting through a hypothalamic relay, later shown to operate via the sympathetic nervous system, it inhibits bone formation, so leptin-deficient mice paradoxically accumulate bone despite their hypogonadism (15, 81). A comparable central route operates for adiponectin, whose central administration also reshapes bone metabolism (82). Adiponectin’s own role is unresolved: it is mitogenic to osteoblasts and inhibits osteoclastogenesis *in vitro*, yet its knockout mice paradoxically show increased bone mass (83). Interpretation is complicated further because marrow adipose tissue is itself an endocrine source of adiponectin that can raise circulating levels as it expands (84). Resistin adds a resorptive input: it is expressed in both osteoblasts and osteoclasts and increases osteoclast differentiation and NF-κB activity (85). The recurring feature of this arm is that no adipokine carries a fixed sign. Each acts differently through central and peripheral routes, and the net skeletal output depends on which route dominates, which is what makes the fat secretome hard to read.

### 4.3 The marrow adipose depot and progenitor fate

The marrow depot is the more direct route, because marrow adipocytes and osteoblasts arise from a shared progenitor whose fate decision is itself a determinant of bone mass. Leptin-receptor-expressing stromal cells are the principal source of osteoblasts, adipocytes, and fibroblast colony-forming units in adult marrow (86). This places bone formation and marrow adipogenesis on one lineage and makes them reciprocal, so that signals pushing the progenitor toward fat tend to withdraw it from bone. That reciprocity is a first approximation rather than a strict zero-sum rule, because parts of the same lineage retain a conditional capacity to form osteoblasts, as Section 4.4 sets out. Leptin acts locally at this branch point. Deletion of its receptor from limb stromal cells increases osteogenesis while decreasing adipogenesis, which indicates that leptin–LepR signaling normally favors fat over bone through Jak2/Stat3 (87). Aging and a high-fat state drive this decision toward adipocytes, expanding marrow fat while impairing the osteogenic and hematopoietic regeneration the niche supports, and the effect of high-fat feeding is amplified in aged animals (16, 88). Pharmacological activation of PPARγ reproduces this shift, as the antidiabetic agent rosiglitazone diverts marrow progenitors toward fat and lowers bone mass (89). The marrow adipose depot therefore does more than correlate with bone loss: by claiming progenitors that would otherwise become osteoblasts, it removes cells from the formation side of the coupled unit. Marrow adipocytes further act on the resorption side, expressing RANKL that supports osteoclast differentiation under inflammatory or glucocorticoid stimulation (90). A related ectopic depot reinforces this resorptive input, since intermuscular adipose expansion drives bone loss through fibro-adipogenic-progenitor-mediated osteoclastogenesis (91).

### 4.4 Heterogeneity of the Adipoq lineage and its conditional osteogenic potential

The account above treats the marrow depot as one compartment, but genetic fate mapping has resolved it into a lineage whose members differ in function. Single-cell transcriptomics of marrow stromal cells identifies a population that expresses adipocyte genes, including Adipoq, yet holds no lipid droplets. These marrow adipogenic lineage precursors, also described as Adipo-CAR cells, are largely non-proliferative, occupy pericytic and stromal positions in a marrow-wide network, and maintain marrow vasculature rather than serving only as a store of fat (92). A smaller and distinct subset co-expresses Adipoq and Osx and lies in the endosteal niche; intersectional fate mapping with two recombinases shows that these adipo-osteoprogenitors give rise both to osteoblasts on trabecular and endocortical surfaces and to Perilipin-positive marrow adipocytes, and that they decline with age in both sexes while being more numerous in young females than in young males (93). Mature lipid-laden adipocytes and the LepR-expressing and CAR populations of Section 4.3 overlap with these subsets without being identical to them, so Adipoq-lineage cells are better read as a hierarchy than as a depot.

The osteogenic capacity of this lineage is conditional rather than fixed, which bears on how the fat arm should be graded. In Adipoq reporter mice, labeling is confined to a few stromal cells at one month but marks the majority of osteoblasts and osteocytes by four to nine months, so the traced contribution to bone rises with age (94). In the same model, GsαR201C and estrogen expose different osteogenic subsets at different skeletal sites, since metaphyseal formation is sustained by traced cells under GsαR201C alone whereas intramedullary formation in the diaphysis requires GsαR201C together with estrogen (94). Fate can also be redirected genetically: Adipoq-lineage progenitors express Shn3 more highly than other mesenchymal populations, and deleting it in these cells shifts them toward osteoblasts, raising trabecular bone mass and improving fracture healing under pathological conditions (95). These results are not uncontested. Early studies using constitutive Adipoq-Cre drivers described these cells as unipotent adipocyte precursors with little or no capacity for osteoblast differentiation, whereas the inducible tracing above found the endosteal subset contributing to both lineages under steady-state conditions; the discrepancy is attributed to heterogeneity within the lineage, to skeletal site, and to the driver used. Adipoq-Cre also leaves periosteal leptin-receptor-positive progenitors untargeted (96). This edge is therefore recorded here as conditional and disputed rather than as an established osteogenic route.

The lineage also does more than supply adiponectin and RANKL. Marrow adipogenic lineage precursors are the dominant marrow source of RANKL, and deleting it in Adipoq-labeled cells raises trabecular bone mass and lowers osteoclast number while leaving cortical bone intact (97). Because that experiment used a constitutive Cre and scored bone from one month of age, it could not separate developmental from adult effects; inducing the same deletion at three months reproduces the phenotype and additionally protects and restores trabecular bone around ovariectomy, which places precursor-derived RANKL at the center of trabecular turnover in the adult skeleton (98). Beyond resorption, these cells support hematopoiesis, help maintain vascular integrity, and participate in regeneration after injury (96).

A recent review in this journal takes these cells as its primary subject and organizes the evidence by their identities, functions, and mechanisms (96). The present synthesis is organized differently, around three secretome arms converging on the remodeling unit, so the Adipoq lineage enters it as source-side heterogeneity within the fat arm rather than as the object of review. Read through that lens, the lineage supplies a concrete instance of the conditional edge signs developed in Section 6.2, since the same cells withdraw progenitors from osteoblast fate under one set of conditions and contribute to osteoblasts under another. The sign carried by the marrow depot therefore depends on the state of the niche, and not on the size of the depot alone.

### 4.5 Lipotoxicity and age-dependent signal inversion

Beyond diverting progenitors, marrow adipocytes act on mature bone cells through the lipids they release. Fatty acids secreted by marrow adipocytes, predominantly palmitate and stearate, impair osteoblast function and survival by inhibiting Runx2 activity and activating caspases in human osteoblasts (99, 100). *In vivo*, this lipotoxicity operates through a fatty-acid–ROS–p53 axis that triggers mitochondrial apoptosis of osteoblasts and lowers bone mass in metabolic disease (101). The consequences of adipokine signaling also change with age. In young bone, adiponectin receptor engagement promotes osteogenesis and suppresses osteoclastogenesis. In aged bone, the same engagement instead induces cellular senescence and impairs osteogenic activity (102), an inversion traced to age- and cell-type-dependent shifts in receptor expression and downstream Wnt, MAPK, and mTOR signaling. This age dependence matters, because the same fat-derived signal can be neutral or protective in youth yet contribute to remodeling imbalance once aging supplies a permissive, inflammatory background.

### 4.6 Human evidence: marrow-fat imaging and circulating adipokines

The clinical traction of the fat arm comes from its measurability, since marrow adipose tissue can now be quantified non-invasively in humans. Magnetic resonance spectroscopy and proton-density fat-fraction imaging show that vertebral marrow fat is higher in osteoporosis (103, 104). Marrow fat fraction also correlates with circulating RANKL in postmenopausal women (105) and, in obese patients undergoing bariatric surgery, higher preoperative fat fraction predicts lower bone mineral density one year later (106), and fat fraction distinguishes patients with osteoporotic vertebral fractures from those without (107). Marrow fat therefore offers a measurable biomarker as well as a mechanism. Multi-parametric approaches extend this readout beyond fat content alone. Simultaneous PET/MR in 110 patients showed that marrow fat fraction varies by skeletal region and relates inversely to glucose uptake and to diffusivity, the fat-to-uptake relation being the stronger of the two (108). In parallel, the CT acquired during 18F-FDG PET/CT can be used opportunistically to read vertebral and femoral-neck attenuation against densitometric thresholds, as shown in patients treated for breast cancer (109). Co-registering metabolic activity, marrow composition, and bone density within one examination is therefore already technically available, although the published cohorts are oncological and their treatments act on the fat and the metabolic axes at once. These measures are best read as indirect, multi-parametric descriptions of the aging marrow milieu rather than as validated readouts of the secretome.

Circulating adipokines add a second layer of human evidence, and it carries the same conflicting signs seen in the mechanistic work. In meta-analyses, serum leptin was on balance positively associated with bone mineral density (110) and was lower in older adults with osteoporosis than in controls of normal density (111), whereas in postmenopausal women adiponectin related inversely to density once body composition was accounted for (112, 113). The fracture data sharpen the adiponectin signal. In older men followed prospectively, high serum adiponectin predicted incident osteoporotic fracture, although its predictive value waned over longer follow-up (114). Frailty and osteoporotic fractures further act as mutually reinforcing risks in aging (115). Human associations therefore point in opposite directions for the two best-studied adipokines, which mirrors at the population level the divided signs their mechanisms predict. Three uncertainties temper the promise. The direction of causality is unresolved, since cross-sectional imaging cannot settle whether marrow fat drives bone loss or accumulates as a consequence of failed osteogenesis. Marrow adipose tissue is also heterogeneous, comprising regulated and constitutive depots that differ in cellular and lipid properties, so a single fat-fraction value may conflate signals of opposite meaning (116). Finally, the conflicting signs of leptin and adiponectin mean that no adipokine can yet be assigned a clean therapeutic direction. What is consistent is that fat and bone are metabolically entangled, and more so with age.

The evidence for this arm is weighted toward models. Naturally aged systems contribute two pieces: the skeletal effect of adiponectin receptor signaling reverses between young and aged mice (102), and marrow fat rises with age in human biopsy and imaging series (79, 103). The central mechanisms come from elsewhere. The leptin pathway was defined in leptin-deficient and genetically obese mice (15, 81), marrow-adipocyte RANKL required glucocorticoid stimulation of human cells (90), the lipotoxicity data derive largely from type 2 diabetes and high-fat feeding (101), and the progenitor fate switch was mapped with conditional genetics and high-fat diet (86, 87). These enter the framework as candidate mechanisms whose operation during unmanipulated aging is not yet shown. Because lipotoxic fatty acids and dysfunctional marrow adipocytes also generate the inflammatory signals that accompany senescence, the fat arm feeds directly into the senescence-associated secretory phenotype and inflammaging.

## 5 Senescence and inflammaging as the amplifier: the SASP arm

### 5.1 Senescence as the hinge of the framework

Cellular senescence is one of the hallmarks of aging (117), and its secretory program provides an important link between aging tissues and the skeleton. Unlike muscle and adipose tissue, senescence is a cellular state that develops within bone as well as in peripheral tissues. In this review, the senescence-associated arm therefore refers specifically to senescent cells within bone and marrow, whereas senescence arising in muscle or adipose tissue is considered within the corresponding tissue sections. The senescence-associated secretory phenotype (SASP) comprises cytokines, chemokines, proteases, and other factors released by these cells (118). Many of these mediators overlap with inflammatory signals associated with muscle and adipose tissue. Inflammaging, the chronic low-grade inflammatory state that accompanies aging (119–122), provides the broader context in which these signals coexist. Evidence relevant to bone includes the accumulation of senescent cells within the skeleton, their effects on remodeling cells, and experiments in which reducing senescent-cell burden attenuates bone loss.

### 5.2 Senescent cells accumulate in aging bone

Senescent cells accumulate within the aging skeleton and act on both arms of remodeling from inside the bone (123, 124). With age, osteocytes, osteoblast progenitors, and marrow immune cells acquire markers of senescence, including elevated p16^Ink4a^ and senescence-associated distension of satellites (SADS). Senescent osteocytes rise several-fold in old cortical bone (17, 125). Aging also expands immunoregulatory T-cell populations, including CD8^+^ subsets implicated in osteoporosis (126).

Human bone carries a related signature, although the evidence is of a different kind. At the level of senescence markers themselves, the human work speaks to cause more than to accumulation: in elderly postmenopausal women, three weeks of estrogen therapy altered neither senescence biomarkers nor the secretory phenotype in bone biopsies, and in young adult mice neither ovariectomy nor orchidectomy induced them, which separates senescence from sex-steroid loss as a distinct lesion (127). A direct comparison of senescence markers between young and old human bone is not established by these studies, so the age-related accumulation itself rests on the murine data above, while human tissue shows the corresponding shift at the proteomic level (Section 5.3). Beyond bone cells, the SASP of osteoporotic osteoblasts alters endothelial cell migration and proliferation *in vitro*, extending its reach to the bone vasculature (128). The human evidence is therefore indirect for accumulation and stronger for the independence of senescence from sex-steroid loss, and none of it assigns causal weight.

Conditioned medium from senescent cells impairs osteoblast mineralization while enhancing the survival of osteoclast progenitors, so the senescent secretome pushes formation down and resorption up at once (18). **Figure 2C** presents the senescence arm as an amplifying background that shares IL-6 and TNF-α with the muscle and fat arms. Proteomic analysis of human bone across the lifespan confirms the pattern at the tissue level: inflammatory pathways are progressively activated while signals promoting bone formation are suppressed (129). Because osteocytes coordinate the remodeling unit, their entry into senescence is especially consequential: the cell that regulates coupling begins to secrete a pro-resorptive, anti-anabolic program. Consistent with this, ablating osteocytes triggers SASP accumulation in marrow progenitors and accelerates skeletal aging (130), and suppression of osteocyte autophagy alone is sufficient to reproduce features of skeletal aging (131).

### 5.3 A shared secretome: amplification, not addition

The integrating feature of this arm is that the SASP is built from the same mediators the muscle and fat arms already use. Its dominant components include interleukin-6, whose pro-inflammatory actions proceed largely through trans-signaling via its soluble receptor (132). They also include tumor necrosis factor-α, the very cytokine through which inflammatory myokines raise resorption (72, 73). The fat arm converges on the same catabolic output, since lipotoxic marrow adipocytes injure osteoblasts through a fatty-acid–ROS–p53 axis (101). Inflammaging is therefore not modeled here as a fourth source arm, and the shared cytokines are entered as source-defined edges rather than once each, so that IL-6 and TNF-α are not counted twice when they appear both as inflammatory myokines and as SASP components. It raises the background concentration of the signals already in play, which lowers the threshold at which catabolic myokines and adipokines act and sustains their effect between acute stimuli. A chronically inflammatory environment may therefore increase the magnitude or duration of otherwise transient tissue-derived signals. Whether these interactions materially amplify age-related remodeling imbalance in humans remains to be established.

### 5.4 The estrogen-deficiency paradigm

The estrogen-deficiency model, long recognized as a central axis of age-related bone loss (133), is a concrete and well-mapped instance of this inflammatory logic. Loss of estrogen at menopause acts on bone in part through direct effects on bone cells, such as the induction of osteoclast apoptosis through an estrogen-receptor-α–Fas ligand axis (134). It also acts indirectly: it enhances the production of TNF-α by T cells, and this T-cell–derived TNF-α augments RANKL- and M-CSF–driven osteoclastogenesis. Accordingly, ovariectomy fails to cause bone loss in animals lacking T cells or TNF (135). In early postmenopausal women, marrow cells bearing RANKL increase as estrogen declines, which links the human syndrome to the same effector (136). The expansion of TNF-producing T cells in the marrow is the proximate lesion (137), and it is sustained because estrogen withdrawal prolongs T-cell lifespan through an IFN-γ–driven increase in antigen presentation (138). Its original investigators framed postmenopausal bone loss as an inflammatory process, not a purely endocrine one (139). It is thus a specific realization of the general principle above: a systemic shift toward inflammation that acts, through RANKL, on the resorptive arm of the coupled unit. The immune contribution to this paradigm is not confined to T-cell TNF-α. Deleting RANKL from B lymphocytes partially protects mice from ovariectomy-induced bone loss in cancellous but not cortical bone, whereas deleting it from T lymphocytes does not, and neither deletion alters bone mass while estrogen is replete (140). The B-cell contribution is nonetheless contested, since mice lacking mature B cells lose bone after ovariectomy much as controls do (141). These immune sources act on the RANKL axis directly, but they are not treated here as a fourth arm of the secretome; they enter the framework as effectors of the inflammatory background defined in Section 1.1. Nearly all of the mechanistic work behind this paradigm used ovariectomy, which removes estrogen abruptly and completely at a chosen moment. Natural aging withdraws it over years and alongside other changes, so the two are not equivalent exposures. The ovariectomy findings therefore enter this framework as candidate mechanisms for the aging skeleton, and the marrow RANKL observations in early postmenopausal women are the part of this paradigm measured during natural reproductive aging (136). Reproductive aging and cellular senescence reach the same node by different routes.

Because this paradigm is defined by the withdrawal of a single hormone, it also marks where the framework becomes sex-specific. Marrow adipose tissue differs between the sexes in a pattern that reverses across the lifespan: vertebral marrow fat rises gradually throughout life in men but sharply in women between 55 and 65 years of age, so that after 60 it stands roughly a tenth higher in women than in men, reversing the sex difference reported below that age (142). An androgen-withdrawal counterpart to the estrogen-loss model exists in men, since androgen-deprivation therapy for prostate cancer accelerates bone loss, and in a cohort of 28 treated men vertebral marrow fat fraction rose over six months while the marrow change tracked the later decline in bone mineral density (143). That series is small, and the association with later bone loss rested on the spectroscopic rather than the Dixon fat-fraction measurement, so the parallel is suggestive rather than established. Individual edge signs may themselves be sex-dependent, as the sex-specific skeletal actions of irisin show (60, 63). Sex is therefore a parameter of this network rather than a covariate to be adjusted away, and **Table 2** records the population and sex behind each human observation.

### 5.5 Causal evidence from senescent-cell clearance

The causal weight of the senescence arm rests on reversal experiments, which set it apart from the largely associative evidence available for individual myokines and adipokines. Eliminating senescent cells in aged mice, whether genetically or with senolytic compounds identified by targeting their apoptosis-resistance networks (144–146), raises bone mass and improves microarchitecture (18). The benefit holds after focal radiation, when senescent-cell burden is pharmacologically reduced (147). Suppressing the SASP alone with a JAK inhibitor reproduces much of that benefit, which supports an important contribution of SASP signaling to bone loss (18); cell-clearance (senolytic) and pathway-inhibition (senomorphic) approaches are not mechanistically equivalent, however, so this comparison points to the secretome without isolating it as the sole cause.

The effect generalizes beyond bone. Genetic clearance of p16^Ink4a^-positive cells delays age-related deterioration across multiple organs and extends healthy lifespan (148, 149), and the same senolytic strategy improves physical function and extends lifespan in old animals (150); JAK inhibition alleviates the SASP and frailty of aging (151), and JAK1/2 blockade mitigates the skeletal phenotype of progeroid mice (152). That an intervention aimed at the secretome corrects the bone phenotype is the strongest available evidence that secretome-driven signaling contributes causally rather than incidentally. The support is partial, however, since genetic clearance of senescent cells is not a specific blockade of the secretome, and JAK inhibition has additional actions, so these experiments support a causal role without proving one. Open questions remain: which senescent cell type is the primary driver in aging human bone, whether the murine reversal translates to patients, and how to avoid the off-target skeletal effects some senolytics carry. Section 7 takes up those therapeutic considerations.

Of the three arms this one carries the most evidence from naturally aged tissue. Senescent osteocytes and their secretory markers were counted in old against young mice without intervening culture (17), and the clearance experiments that give the arm its causal weight were performed in aged animals with established bone loss (18). Two qualifications attach. The estrogen-deficiency paradigm of Section 5.4 rests on ovariectomy, and part of the clearance evidence comes from focal irradiation and progeroid models (147, 152), in which senescence is induced or accelerated instead of accumulating over time. The human contribution is narrower than the murine one, since it establishes the independence of senescence from sex-steroid status (127) and a proteomic shift with age (129) without demonstrating marker accumulation across the human lifespan. This arm is therefore the best evidenced in natural aging and still short of human causal proof. Together, these findings support considering inflammaging as a shared context in which muscle-, fat-, and senescence-associated signals may interact, a relationship examined further in Section 6.

## 6 An integrated secretome–remodeling network

### 6.1 Nodes, edges, and the inflammaging background

The evidence reviewed above can be summarized as a secretome–remodeling network linking muscle, adipose tissue, and senescent cells to the bone remodeling unit. The term remodeling is used deliberately at this level because, as **Table 1** shows, most of the relationships identified here are supported by effects on formation or resorption rather than by direct evidence that a resorption-to-formation coupling signal has been altered. Coupling is therefore retained for the anatomical and physiological unit on which these signals converge. Within the network, muscle, adipose tissue, and senescent cells constitute the principal signal sources, while their secreted mediators form the relationships through which they influence osteoblasts, osteoclasts, and osteocytes. These relationships are defined not only by the mediator itself but also by its source, temporal pattern, and receptor route. IL-6 illustrates this distinction: acute release from contracting muscle, sustained production during chronic inflammation, and release as part of the senescence-associated secretory phenotype represent biologically different contexts, and the source-defined skeletal relationships are treated separately in **Table 1**. Inflammaging is considered differently from the three source compartments. Rather than constituting a fourth arm, it represents a shared inflammatory context that may modify the magnitude or direction of several source-specific effects and prevents overlapping cytokines from being counted as independent signals. Current evidence supports this role as a contextual modifier, but does not allow its contribution to be quantitatively parameterized. Although the review focuses primarily on signals directed toward bone, communication is not exclusively one-way. Bone-derived osteocalcin, for example, promotes myofiber adaptation to exercise and declines with age (153), illustrating a reverse bone-to-muscle relationship. **Figure 3** summarizes these relationships with the remodeling unit at the center, the three principal sources around it, and inflammaging as the shared background. Edge width is kept uniform because relative effect sizes cannot yet be assigned reliably; solid lines indicate direct mechanistic evidence, whereas dashed lines denote relationships that are context-dependent or not consistently established.

**Figure 3:**
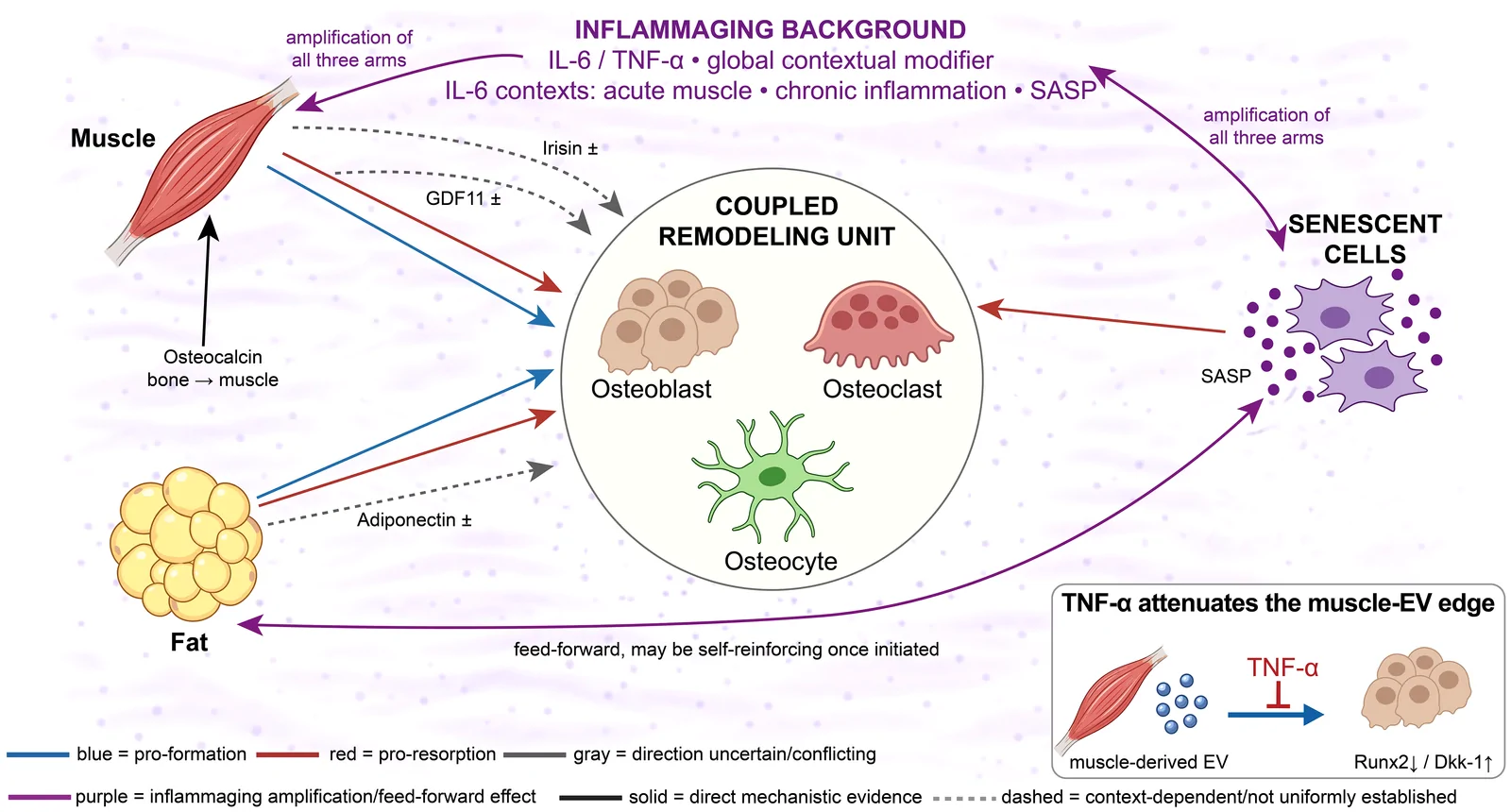
An integrated secretome–remodeling network with inflammaging as a global contextual modifier. The three arms are integrated into a single network in which nodes are the cells of the remodeling unit (osteoblast, osteoclast, osteocyte) and edges are secreted factors reaching them from the muscle, adipose, and senescent-cell arms. Each edge is defined by its source, temporal pattern, and receptor route. IL-6 is shown in three biological contexts—acute muscle release, chronic inflammation, and the senescence-associated secretory phenotype—because the same molecule may carry different implications across source and timing. Edge color encodes direction (blue, pro-formation; red, pro-resorption; gray, direction uncertain or conflicting), whereas purple denotes inflammaging-mediated amplification and feed-forward effects. Solid lines indicate direct mechanistic evidence; dashed lines denote context-dependent or not uniformly established relationships. Inflammaging (IL-6, TNF-α) is treated as a global contextual modifier that may alter the strength or direction of multiple secretome-mediated edges rather than as a single edge or conventional node. A feed-forward loop, in which senescent cells sustain inflammaging that in turn amplifies signals across the three arms and promotes further senescence, may render age-related bone loss self-reinforcing once initiated. Edge sign remains conditional for irisin, adiponectin, and GDF11, reflecting state dependence and heterogeneity in dose, model, assay, and sampling. A representative cross-arm interaction is highlighted, in which TNF-α attenuates or abolishes the osteogenic output of the myogenic extracellular-vesicle edge through Runx2 suppression and Dkk-1 induction; a reverse bone-to-muscle edge (osteocalcin) is also shown. The complete edge list, with factors, sources, target cells, directions, evidence for direct cellular action and resorption-to-formation coupling, the effect level at which each edge is evidenced, and references, is given in **Table 1**. Almost every edge shown here is evidenced at the level of one arm of remodeling, or of both arms independently, rather than at the level of a coupling signal, which is why the network is named for remodeling rather than for coupling.

### 6.2 Conditional edge signs and cross-arm interactions

A defining property of the network is that its edges carry conditional signs, not fixed ones. This conditionality may reflect genuine state dependence, although heterogeneity in dose, model, assay, and sampling time cannot be excluded. Irisin, adiponectin, and GDF11 each appeared in earlier sections with opposing reported effects. In a network view this is expected, because the sign of an edge depends on the state of the node it enters and on the inflammatory background. Inflammaging sets that background for many, though not necessarily all, edges. By raising the ambient concentration of IL-6, TNF-α, and related cytokines, senescence changes the weight of the muscle and fat edges. The same myokine or adipokine input may therefore yield a larger catabolic output in an aged, inflamed marrow than in a young one. The interaction can be shown at the level of individual edges. Tumor necrosis factor-α blunts the osteogenic effect of muscle-derived extracellular vesicles, which shows that an inflammatory signal can attenuate or abolish the osteogenic output of a myokine channel (154). The same cytokine also acts directly on the formation node, where it suppresses osteoblast differentiation through inhibition of Runx2 (155, 156) and induces the Wnt antagonist Dkk-1 to tip remodeling toward resorption (157). Inflammaging does more than weaken single edges; it also amplifies the resorptive channel it shares with the arms. Tumor necrosis factor-α and RANKL converge on overlapping signaling in osteoclast precursors, so that TNF-α acting through its type 1 receptor markedly enhances RANKL-driven osteoclastogenesis rather than adding to it independently (158). In inflammatory arthritis, an analogous amplification operates, where IL-34-driven synoviocyte-macrophage crosstalk promotes RANKL-dependent osteoclastogenesis and bone erosion (159). In an inflamed, senescent marrow this synergy means that a RANKL signal generated within bone, or induced downstream by peripheral inflammatory inputs, is read out as a larger resorptive response. The conditional sign of an edge therefore depends on the factor itself and on the inflammatory background that inflammaging maintains.

**Table 1** summarizes the factors, their sources, their cellular targets, and the direction of their effects, and serves as the edge list of the network. For each edge it records the effect on formation and on resorption, whether the evidence bears directly on coupling or is inferred from net changes in bone mass, and the type of experimental evidence available, so that the strength of each claim is visible alongside its direction. **Table 2** consolidates the human evidence for the three arms, so that the mechanistic edges can be read against what has been measured in people. The same logic applies to differences between patients, not only between factors. The sign and weight of many edges depend on sex, on menopausal stage, on skeletal site, and on whether bone loss is of the early high-turnover or the later low-turnover type. Early postmenopausal loss is dominated by a high-turnover, estrogen-withdrawal state, whereas later age-related loss is often low-turnover and formation-poor, and the cortical and trabecular compartments do not respond identically. These state variables are part of the network’s specification and not noise around a single trajectory, and any quantitative version of the model will have to carry them as conditions on each edge.

### 6.3 Integrative implications of the network

Considering these relationships together highlights connections that are less apparent when individual mediators are examined in isolation. Shared signaling between muscle and bone, together with the inflammatory changes that accompany aging, may contribute to the frequent coexistence of osteoporosis and sarcopenia. The same perspective may also help account for variation in the effects of interventions directed at individual mediators, since other signaling pathways and the surrounding inflammatory state remain active when one pathway is altered. Because signals from different sources ultimately influence the same remodeling cells and pathways, distinct upstream perturbations can also produce overlapping skeletal outcomes.

This organization differs from previous crosstalk reviews that primarily catalog myokines, osteokines, and adipokines as parallel groups of mediators (20, 160) or follow individual pathways between tissues (161). By considering the source, target, and biological context of each relationship together, it also raises the possibility of interactions across the three compartments. One such possibility is a feed-forward interaction between cellular senescence and chronic inflammation, in which inflammatory signaling enhances the effects of tissue-derived mediators while senescent cells help sustain the inflammatory environment. Whether such a loop contributes to the progression of age-related bone loss remains uncertain. The available studies differ substantially in dose, experimental model, assay, and sampling time, and these sources of heterogeneity currently prevent the relative contribution of this interaction from being established.

### 6.4 Limits and the shift to a therapeutic question

The model remains qualitative. The direction and relative contribution of many relationships are uncertain and are derived largely from rodent studies, and the network has not been parameterized in human bone. It should therefore be regarded as an organizing hypothesis rather than a quantitative circuit. Because the present framework focuses primarily on signals directed toward the remodeling unit, reverse bone-to-muscle relationships remain outside its current scope. One practical implication is that therapeutic approaches can be distinguished by whether they act on a defined pathway, as with sclerostin or activin receptor blockade, or on broader senescence- and inflammation-related processes, as attempted with senolytic and anti-SASP strategies. The latter may influence several pathways simultaneously, whereas pathway-specific interventions are likely to exert more selective effects. Whether broader targeting produces greater or more durable skeletal benefit, however, remains to be established in humans. Section 7 considers the available interventions in light of these differences in mechanistic scope and translational evidence.

## 7 Therapeutic implications

### 7.1 Three levels of intervention

Therapeutic approaches discussed in this section differ in both their biological scope and the maturity of the supporting evidence. Pathway-specific treatments, including agents directed at sclerostin, RANKL, PTH signaling, or activin type II receptors, act on relatively defined components of bone or muscle regulation. Senolytic and senomorphic approaches target cellular senescence or its secretory phenotype and may therefore influence a broader inflammatory environment. Exercise differs from both pharmacological strategies by simultaneously affecting mechanical loading, muscle-derived signals, adipose tissue, and systemic inflammation. **Figure 4** compares these approaches according to their mechanistic breadth and current level of translational evidence. The corresponding evidence levels, mechanistic breadth, and key limitations of representative interventions are summarized in **Table 3**. Notably, the interventions with the strongest human evidence are predominantly pathway-specific, whereas approaches directed at senescence and the broader inflammatory environment remain at an earlier stage of clinical development.

**Figure 4:**
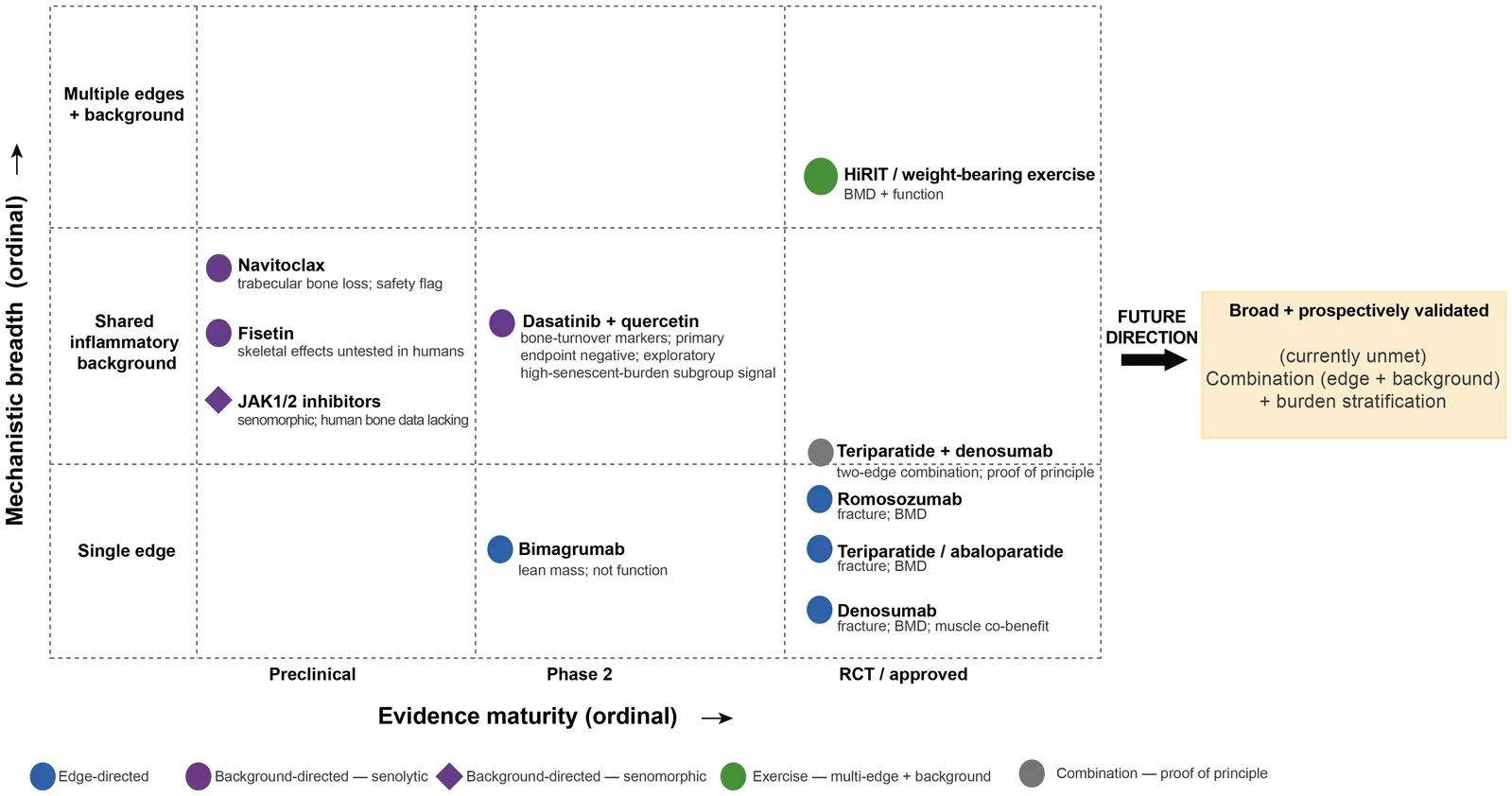
Edge-, background-, and exercise-directed strategies mapped as an evidence-maturity matrix. Interventions are positioned in two dimensions: overall evidence maturity (x-axis: preclinical, phase 2, then randomized controlled trial or approved) against the breadth of mechanistic action (y-axis: single edge, shared inflammatory background, then exercise acting on multiple edges plus the background). Both axes are assigned by explicit rules. On the evidence axis, preclinical denotes agents supported only by cell or animal work; phase 2 denotes agents with controlled human trial data short of a pivotal trial; and randomized controlled trial or approved denotes agents with a pivotal trial powered on fracture or bone mineral density, or with regulatory approval for a skeletal indication. Placement follows the highest level reached, irrespective of whether the result was positive, so an agent whose pivotal trial missed its primary endpoint is positioned by the trial and not by its outcome. On the breadth axis, single edge denotes action on one defined edge of the network; shared background denotes action on the IL-6 and TNF-α inflammatory background common to the three arms; and multiple edges plus background denotes concurrent action on several edges and on that background. The teriparatide + denosumab combination is shown between the single-edge and shared-background levels as a two-edge proof of principle; it does not represent background-directed therapy. The axes are ordinal rather than continuous, they are not commensurable with one another, and the primary outcome behind each placement (bone mineral density, bone-turnover marker, lean mass, or physical function) is noted beside the agent, since agents are not evidenced on identical endpoints. Horizontal position therefore reports how far an agent has been tested and not how large its skeletal effect is, and vertical position reports mechanistic reach and not therapeutic value; neither axis ranks agents by efficacy, and agents evidenced on different endpoints should not be compared directly. Edge-directed agents (blue: romosozumab, teriparatide, abaloparatide, denosumab, bimagrumab) have the firmest human data but each acts on a single channel; bimagrumab increases lean mass without restoring skeletal function after hip fracture, illustrating that adding mass is not the same as restoring the network. Background-directed agents, comprising senolytics (dasatinib plus quercetin, navitoclax, fisetin) and senomorphic JAK inhibition (purple), carry the strongest conceptual claim yet remain early and agent-specific: the first controlled bone trial of dasatinib plus quercetin was negative overall, with only an exploratory high-senescent-burden subgroup signal, and navitoclax causes trabecular bone loss. Exercise (green: high-intensity resistance and impact training) is the broadest strategy, acting on multiple edges and the shared background, with acceptable safety in screened, supervised populations; its efficacy and safety remain population- and protocol-dependent, and the trials that established it screened and supervised their participants, so it remains untested in frail older adults who would gain the most. No single agent covers the whole network; the arrow indicates the unmet direction toward an edge-plus-background combination with burden stratification that would be both broad and prospectively validated. Evidence grades, breadth, and safety signals for each agent are detailed in **Table 3**.

**Table 3:** Evidence and limitations of edge-, background-, and exercise-directed strategies.

Strategy (level)	Representative agent	Target	Highest human evidence	Breadth	Framework limitation/selected safety consideration	Ref
Edge — formation	Romosozumab	Sclerostin (Wnt brake)	RCT/approved (FRAME, ARCH)	Narrow	Single edge; bypassable	(162, 163)
Edge — formation	Teriparatide/abaloparatide	PTH/PTHrP signaling	RCT/approved (ACTIVE)	Narrow	Single edge	(164, 165)
Edge — resorption	Denosumab	RANKL	RCT/approved (FREEDOM)	Narrow (shared edge)	Muscle co-benefit but single channel	(166, 168)
Edge — muscle	Bimagrumab	Activin type II receptor	Phase 2 and Phase 2a/b RCTs	Narrow	Lean mass but no functional gain after hip fracture	(170, 172, 173)
Background — senolytic	Dasatinib + quercetin	Senescent cells (clearance)	Phase 2 (primary endpoint negative; subgroup only)	Broad (concept)	Exploratory signal in a high-burden subgroup; not prospectively confirmed	(18, 150, 175)
Background — senolytic	Navitoclax	Senescent cells (clearance)	Preclinical	Broad (concept; agent-specific)	Trabecular bone loss, impaired osteoprogenitors	(176)
Background — senolytic	Fisetin	Senescent cells (natural)	Preclinical (health/lifespan)	Broad (concept)	Skeletal effects untested in humans	(177)
Background — senomorphic	JAK1/2 inhibitors	SASP (pathway inhibition)	Preclinical (frailty, progeroid)	Broad (SASP modulation)	Human bone data lacking	(151, 152)
Exercise	HiRIT/weight-bearing	Multiple edges + background	RCT (LIFTMOR); semi-randomized (LIFTMOR-M)	Broad; safety protocol-dependent	Compliance high but cohorts screened and supervised; untested in frail elderly	(178, 180, 182, 183)
Combination (proof of principle)	Teriparatide + denosumab	Two edges	RCT (DATA)	Intermediate (two edges)	Two-edge proof of principle only; does not test background-directed therapy	(184)

Comparison of the three strategy levels plus a combination proof of principle mapped in **Figure 4**. Each row gives a representative agent, its molecular target, the highest level of human evidence, mechanistic breadth, the principal framework limitation with a selected safety consideration where one is material, and supporting references. This column is not a systematic safety review of the agents listed. The evidence and breadth entries follow the same explicit criteria as the two axes of **Figure 4**, where they are defined; both are ordinal, and ‘highest human evidence’ records the highest level of testing an agent has reached rather than the size or direction of its effect, so agents listed at the same level are not thereby equivalent in benefit. Because the agents in this table are evidenced on different primary outcomes, including fracture, bone mineral density, bone-turnover markers, lean mass, and physical function, the rows are not directly comparable with one another and no ranking by efficacy is implied. Strategy level is color-coded: edge-directed (blue), background-directed (purple), exercise (green), and combination (gray). Note the inverse pattern between evidence maturity and mechanistic breadth in the strategies mapped here: the agents at the most mature evidence levels act on relatively narrow targets, whereas broader strategies remain earlier in translation. Negative or subgroup-only trial results (dasatinib plus quercetin) and skeletal safety signals (navitoclax) are reported as found. Numbers in parentheses refer to the numbered reference list. JAK, Janus kinase; PTH, parathyroid hormone; PTHrP, parathyroid hormone-related protein; RANKL, receptor activator of nuclear factor-κB ligand; RCT, randomized controlled trial; SASP, senescence-associated secretory phenotype.

### 7.2 Edge-directed therapies: mature but narrow

Edge-directed therapies currently have the strongest clinical evidence, although their effects are generally confined to relatively defined signaling pathways. Romosozumab, an antibody against sclerostin, releases the Wnt brake on formation and lowers fracture risk in postmenopausal osteoporosis (162). Its benefit is maintained when it is followed by an antiresorptive (163). Romosozumab therefore provides an example of a relatively pathway-specific intervention on the bone-formation arm. Teriparatide targets the same formation arm by a different route: it builds bone through intermittent activation of PTH signaling and likewise lowers fracture risk (164), an effect shared by the related analog abaloparatide (165). On the resorption side, denosumab, a monoclonal antibody to RANKL, reduces vertebral and non-vertebral fractures in postmenopausal osteoporosis (166), although network meta-analysis places it below parathyroid hormone receptor agonists and romosozumab for clinical fractures (167). RANKL inhibition also improves muscle strength and insulin sensitivity, which suggests that this edge is shared between the bone and muscle nodes of the network (168, 169). On the muscle side, bimagrumab blocks activin type 2 receptors and reliably increases muscle mass, both in chronic disease (170, 171) and in metabolic settings where it also reduces fat (172). The limits of edge-directed action are visible in the same program. In older adults recovering from hip fracture, bimagrumab increased lean mass but did not improve mobility or lower-extremity function (173). The dissociation between increased lean mass and unchanged mobility or lower-extremity function suggests that gains in muscle mass alone may be insufficient to restore functional outcomes in this population.

### 7.3 Background-directed therapy: preclinical rationale and early human evidence

Background-directed therapy targets cellular senescence or its associated secretory and inflammatory processes rather than a single skeletal signaling pathway. It comprises two mechanistically distinct classes: senolytics, which clear senescent cells, and senomorphics or anti-SASP agents, which suppress the secretory phenotype without killing the cell. The two differ in target, safety, and skeletal effect, so results from one should not be generalized to the other. Its preclinical case is strong, while its human case is still forming. In aged mice, clearing senescent cells or suppressing the SASP raises bone mass and improves microarchitecture (18), and the same senolytic approach improves physical function and lifespan (150) and attenuates immune, inflammatory, and metabolic hallmarks of aging across tissues (174). Translation to humans has so far been modest. A phase 2 trial of intermittent dasatinib plus quercetin in postmenopausal women did not meet its primary endpoint of reduced bone resorption. It produced only a transient rise in a formation marker, and exploratory analyses suggested benefit in the subgroup carrying a high senescent-cell burden, a hypothesis that requires prospective validation (175). Safety adds a further caution: acting on the background does not guarantee skeletal safety. The senolytic navitoclax clears senescent cells, yet it causes trabecular bone loss and impairs osteoprogenitor function in aged mice, unlike genetic clearance or JAK inhibition, which preserve bone (176). Other natural senolytics such as fisetin extend health and lifespan with a favorable safety profile in preclinical models (177), though senolytic doses, skeletal efficacy, and safety in humans remain to be established. The available evidence supports skeletal effects of senescence-targeting strategies in preclinical models, but their translation to humans remains uncertain and appears to depend on the specific agent.

### 7.4 Exercise: multi-level effects, with efficacy and safety that depend on population and protocol

Exercise differs from the pharmacological strategies discussed above because it simultaneously affects mechanical loading, muscle-derived signals, marrow adiposity, and systemic inflammation. Mechanical loading suppresses osteocyte sclerostin and drives formation. Contractile activity raises anabolic myokines such as irisin (178), although the acute response depends on protocol and one crossover trial in young adults recorded a fall in circulating irisin immediately after training (179). Habitual activity reduces marrow adiposity, and weight-bearing exercise such as running is associated with lower vertebral marrow fat in humans (180). Regular exercise also lowers systemic inflammatory tone (181). In older adults, high-intensity resistance and impact training improves bone mineral density and physical function, in postmenopausal women (182) and in men with low bone mass (183). These trials were conducted in screened, supervised participants, so their favorable safety profile applies to that setting rather than to every frail older adult. Its mechanism and dose–response remain context-dependent, and its main current limitation is delivery. Whether the intensity required for skeletal benefit can be delivered safely and effectively to frail older adults remains uncertain, because the trials demonstrating benefit were conducted in screened and supervised participants.

### 7.5 Toward combination and biomarker-guided treatment

The three therapeutic approaches differ substantially in both mechanistic scope and the maturity of their supporting evidence. Pathway-specific agents have progressed furthest clinically, whereas interventions directed at cellular senescence or the broader inflammatory environment remain at an earlier stage of translation. Exercise affects several tissues and signaling processes simultaneously, but the available intervention trials were conducted primarily in selected and supervised populations. At present, no validated biomarker identifies patients in whom secretome-associated signaling makes a major contribution to skeletal decline. Combination therapy is nevertheless of interest, since teriparatide plus denosumab increases bone mineral density more than either agent alone (184), although this finding does not establish that combining pathway-specific treatment with senescence- or inflammation-directed therapy would produce the same benefit.

The exploratory association between high senescent-cell burden and response to dasatinib plus quercetin provides a rationale for prospective evaluation of senescent-cell burden as a potential treatment-response marker (175). Circulating extracellular vesicles, their microRNA cargo, and soluble SASP components are additional candidate readouts, but their relationship to skeletal outcomes requires validation. If such biomarkers can be shown to identify biologically distinct patient groups, they could subsequently be incorporated into trials evaluating combinations of pathway-specific and senescence- or inflammation-directed interventions. No controlled trial has yet demonstrated that these biomarkers identify responders, and no trial has established the proposed combination strategy. Biomarker-guided treatment should therefore remain a research hypothesis rather than a basis for current clinical decision-making.

One widely used drug class sits outside this scheme yet bears directly on it. Incretin-based therapies produce substantial weight loss, and weight loss itself withdraws mechanical and endocrine support from bone. In a 52-week placebo-controlled trial in 64 adults at increased fracture risk, once-weekly semaglutide lowered bone mineral density at the lumbar spine and total hip and raised a resorption marker without a matching rise in formation; the investigators attributed the rise in resorption to the accompanying weight loss rather than to a direct skeletal action (185). No trial has yet tested these agents against a fracture endpoint, and whether they alter marrow adipose tissue in humans has not been measured, so their relation to the marrow-fat evidence of Section 4 remains inferential. Whether marrow adipose tissue contributes to the skeletal effects of weight loss, beyond the effects of reduced body mass itself, remains uncertain and has not yet been tested directly in humans.

## 8 Discussion, conclusions and future perspectives

The evidence reviewed here supports substantial signaling between aging muscle, adipose tissue, senescent cells, and the bone remodeling unit. These pathways do not yet establish a single causal mechanism for age-related bone loss, but they converge on osteoblasts, osteoclasts, osteocytes, and inflammatory pathways that regulate remodeling. The term aging secretome is used here to describe this convergence while preserving the distinction between functional remodeling imbalance and direct disruption of coupling signals. Chronic inflammation may modify several of these relationships, although its contribution relative to individual tissue-derived factors remains difficult to quantify in humans. This interpretation is consistent with the frequent coexistence of osteoporosis and sarcopenia and with the variable skeletal effects reported for several individual mediators, but neither observation by itself establishes the proposed mechanism. The exploratory association between senescent-cell burden and response to senolytic treatment further suggests a possible role for biological stratification, although this finding requires prospective validation before it can inform treatment selection.

The scope of this review is intentionally selective. The three arms were selected for coordinate change with skeletal aging and direct signaling to the remodeling machinery, not because they exhaust the secreted inputs bone receives. Bone also participates in endocrine axes with the brain, kidney, liver, pancreas, gonads, vasculature, and immune system, and it returns signals of its own (186). Several enter locally, since IGF-1, androgens and their aromatization, and the OPG–RANKL axis are treated where they bear on the three arms, and osteocalcin appears as a reverse edge in **Table 1**. Others are absent: FGF23–Klotho signaling and chronic kidney disease mineral disturbance, gut-derived serotonin and the incretins, amylin, hypothalamic neuropeptide Y and melanocortin signaling beyond the sympathetic outflow of Section 4.2, and osteokine outputs beyond osteocalcin, such as the Schnurri-3–SLIT2 route by which bone acts on adipose tissue (187). Also absent are the pituitary growth hormone axis upstream of the local IGF-1 actions noted above, circadian and melatonin signaling, and the osteogenic programs shared with vascular calcification. These pathways fall outside the present scope because the review focuses on muscle-, adipose-, and senescence-associated signals converging on skeletal remodeling; their exclusion does not imply lesser biological importance.

Several limitations currently prevent the proposed network from being treated as a quantitative model of human skeletal aging. Most causal evidence comes from rodent genetics or pharmacological manipulation, and the direction and relative contribution of many relationships in aging human bone remain uncertain. Some mediators, including irisin, adiponectin, and GDF11, also show context-dependent or conflicting skeletal effects, which are likely to reflect differences in dose, experimental model, cell type, and physiological state. Resolving these relationships will require better characterization of the relevant cell populations in human bone, particularly through single-cell and spatial approaches, together with prospective evaluation of circulating extracellular vesicles and soluble SASP components as potential readouts of the skeletal aging environment. Integrating tissue, circulating, imaging, and multi-omic data may eventually require computational approaches, including machine-learning methods, but their value will depend on external validation and biological interpretability.

These limitations also constrain therapeutic interpretation. Interventions directed at senescent cells, individual myokines or adipokines, or broader physiological processes such as exercise may each provide information about different components of the proposed network, but none currently validates the network as a whole. In particular, the available evidence does not establish that single-factor strategies are insufficient, nor does it show that combining pathway-specific treatment with interventions directed at senescence or inflammation will improve clinical outcomes. Senescent-cell burden and secretome-related biomarkers therefore remain candidate stratification tools rather than established guides to treatment. Prospective studies will be needed to determine whether such markers identify biologically distinct patient groups and whether they can be used to design or test combination strategies. Until then, the aging secretome should be regarded as a framework for organizing and testing cross-tissue relationships rather than as a predictive clinical model.
