# Supplementary material for: The aging secretome as a candidate driver of bone remodeling imbalance: myokines, adipokines, and senescence-associated secretory factors
Source: Front Endocrinol (Lausanne). 2026 Oct 26;17:1982491. doi: 10.3389/fendo.2026.1982491 (PMC13746441; doi:10.3389/fendo.2026.1982491)
Supplement: Supplementary file 1 [file Table1.docx]

**Supplementary Table S1. Study characteristics, limitations and aging context of the evidence underlying Table 1 and Table 2**

| **Ref** | **Factor or topic** | **Study model** | **Population or system** | **Skeletal outcome measured** | **Major limitation** | **Aging context** |
| --- | --- | --- | --- | --- | --- | --- |
| （13） | Myostatin to osteocyte exosomal miR-218 | Cell | Ocy454 osteocytes and MC3T3 osteoblastic cells (mouse lines) | Osteoblastic differentiation, Runx2, Wnt signaling | In vitro with immortalized lines only; no in vivo or aged-tissue confirmation | Not aging-specific |
| （52） | GDF11 and myostatin; follistatin inhibition | Animal genetic | Gdf11-null mice and follistatin-overexpressing mice | Bone mass, osteoblast and chondrocyte maturation, osteoclastogenesis, fracture | Germline loss cannot separate developmental from adult effects; Gdf11 null is perinatally lethal | Not aging-specific (germline null) |
| （53） | GDF11 lowers bone mass | Animal pharmacological and genetic | Young and aged mice; estrogen-deficiency model; GDF11 treatment and blockade | Bone mass, osteoclastogenesis, osteoblast differentiation, bone regeneration | Exogenous dosing may exceed physiological exposure; benefit of blockade shown against an ovariectomy stimulus | Natural aging (aged mice); aging-adjacent (ovariectomy) |
| （54） | GDF11 inhibits bone formation via Smad2/3 | Cell and animal pharmacological | Mouse bone-marrow mesenchymal stem cells in vitro; intraperitoneal GDF11 in mice | Osteoblastic differentiation; bone formation; age-related bone loss | Systemic injection does not localize the effect; no osteoclast or resorption effect detected | Natural aging (age-related loss accelerated) |
| （14） | Irisin raises cortical bone mass | Animal pharmacological | Young male mice, low cumulative weekly dose of recombinant irisin | Cortical bone mass, tissue mineral density, bending strength; trabecular compartment spared | Young males only; single dosing regimen; no aged animals; cortical effect is partly modeling | Not aging-specific (young mice) |
| （59） | Irisin acts through alphaV integrin receptors | Animal genetic and cell | FNDC5-ablated mice; ovariectomy-induced osteocytic osteolysis | Osteocyte survival, sclerostin production, bone loss | Ablation is constitutive; the skeletal readout depends on the ovariectomy stimulus | Aging-adjacent (ovariectomy) |
| （61） | Irisin stimulates osteoclastogenesis and resorption | Cell and animal genetic | C57BL/6J marrow progenitors in vitro; Fndc5-transgenic mice at three ages | Osteoclast differentiation, resorption on several substrates, bone mass | Forced expression gives supraphysiological exposure; direction opposes the anabolic reports | Not aging-specific (transgenic, cell) |
| （62） | Serum irisin and prevalent fracture | Human observational | 36 overweight postmenopausal women with vertebral fracture and 36 without | Prevalent vertebral osteoporotic fracture; lumbar and femoral BMD; body composition | Small, cross-sectional, overweight-only; no relation to BMD itself; no causal inference | Natural aging (human, postmenopausal) |
| （10） | Matrix IGF-1 maintains bone mass | Animal genetic and human observational | Pre-osteoblastic Igf1r-knockout mice; aged versus young rats; human marrow in age-related osteoporosis | Bone mass, mineral deposition rate, mesenchymal stem cell differentiation, marrow IGF-1 concentration | Human component is a concentration comparison only; the rescue experiment was done in rats | Natural aging (aged rats; older human marrow) |
| （64） | Osteoblast IGF-1 receptor in reloading | Animal genetic with mechanical model | Osteoblast-specific Igf-1r-deficient mice; 14 days hindlimb unloading then 16 days reloading | Periosteal and endosteal bone formation, cortical cross-sectional area | Disuse and reloading is a mechanical model rather than aging; the periosteal response is modeling, not remodeling | Aging-adjacent (unloading and reloading) |
| （65） | FGF2 disruption | Animal genetic | Fgf2-null adult mice; bone-marrow stromal cultures | Trabecular architecture and bone volume, mineral apposition and bone formation rates | Germline null; developmental contribution not separable from adult function | Not aging-specific (germline null) |
| （66） | BAIBA as an osteocyte survival factor | Animal pharmacological and cell | Mice given BAIBA in drinking water; hindlimb unloading model; aged animals | Bone loss, osteocyte apoptosis, muscle function | Protection shown against a disuse stimulus; the aging result is loss of response, with reduced Mrgprd expression as the inferred cause | Aging-adjacent (unloading); natural aging (response lost with age) |
| （67） | Myoblast extracellular vesicle miR-27a-3p | Cell | C2C12 myoblast exosomes applied to MC3T3-E1 pre-osteoblasts | Osteogenic differentiation; APC and beta-catenin signaling | Cell lines only; no in vivo delivery, no aged tissue, no demonstration of bone targeting | Not aging-specific |
| （70） | Muscle extracellular vesicles suppress osteoclast formation | Cell | C2C12-derived vesicles on mouse marrow cells and RAW264.7 cells | Osteoclast formation, TRAP and cathepsin K expression, mitochondrial biogenesis | In vitro only; no effect on primary osteoblasts; no in vivo confirmation | Not aging-specific |
| （71） | Vesicular miR-196a-5p | Cell | C2C12 vesicles; ST-2 and MC3T3-E1 cells; mouse marrow cells | Osteoclast-like cell formation; osteoblastic differentiation | Cell lines only; inference rests on mimic experiments | Not aging-specific |
| （72） | IL-6 requires IL-6 receptor on osteoblastic cells | Cell co-culture and transgenic | Mouse marrow and osteoblastic co-cultures; human IL-6 receptor transgenic mice; dexamethasone | Osteoclast differentiation | Effect requires soluble receptor or dexamethasone; the receptor-transgenic system is artificial | Not aging-specific |
| （73） | IL-7 and TNF-dependent osteoclastogenesis | Human observational and cell | 50 male patients with solid tumors, with and without bone lesions; peripheral mononuclear cell cultures | Osteoclastogenesis; serum IL-7 concentration | Malignancy-driven and male-only; not an aging cohort; serum marker plus ex vivo assay | Disease-associated (solid tumors) |
| （77） | Serum myostatin and BMD | Human observational | 175 healthy Chinese postmenopausal women aged 51 to 75 years | BMD by DXA at several sites, body composition, turnover markers | Null result: no group difference after age adjustment and no correlation with BMD; cross-sectional; single cohort; serum may not reflect local exposure | Natural aging (human, postmenopausal) |
| （78） | Serum myostatin and irisin in rheumatoid arthritis | Human observational | 182 patients with rheumatoid arthritis and 142 controls; sex composition not reported | Sarcopenia status; osteoporotic fracture; skeletal muscle mass index | Disease cohort; cross-sectional; findings come from separate subgroup comparisons rather than a joint analysis | Disease-associated (rheumatoid arthritis) |
| （15） | Leptin acts through a hypothalamic relay | Animal genetic with central infusion | Leptin-deficient and leptin-receptor-deficient obese hypogonadic mice; intracerebroventricular leptin | Bone formation, bone mass | Monogenic obesity model; the central route means this is not a direct action on bone | Not aging-specific (leptin-deficient and obese mice) |
| （81） | Leptin acts via the sympathetic nervous system | Animal pharmacological | Leptin-deficient, wild-type, and ovariectomized mice; beta-adrenergic agonist and antagonist | Bone mass, osteoblast proliferation | Pharmacological adrenergic manipulation; ovariectomy supplies the bone-loss stimulus | Not aging-specific; aging-adjacent (ovariectomy) |
| （82） | Central adiponectin administration | Animal genetic with central infusion | Adiponectin-knockout and wild-type mice on normal diet; intracerebroventricular adiponectin; neurons and marrow stromal cells | Trabecular structure and mineralization, marrow adiposity, osteoclast number, osteogenic markers | Central administration is non-physiological; knockout studied on normal diet only | Not aging-specific (knockout) |
| （83） | Adiponectin in vitro and in vivo | Cell and animal genetic | Primary rat and human osteoblasts; osteoclastogenesis assays; adiponectin-knockout male mice at 14 weeks | Trabecular bone volume and number, osteoblast mitogenesis, osteoclastogenesis | In vitro and in vivo directions conflict, which the authors attribute to indirect actions; young male mice only | Not aging-specific (knockout, cell) |
| （102） | Aging alters adiponectin receptor signaling | Animal (aged versus young) and cell | Young and aged mouse bone marrow; AdipoRon treatment; single-cell sequencing | Bone regeneration and repair, osteogenesis, osteoclastogenesis, cellular senescence | Agonist-based rather than physiological; repair model rather than steady-state remodeling | Natural aging (young versus aged mice) |
| （85） | Resistin in bone cells | Cell | RAW264.7 and MC3T3-E1 lines; primary human marrow stem cells and mature osteoblasts | Osteoclast number, NF-kB promoter activity, osteoblast proliferation | Expression and in vitro response only; no in vivo or human skeletal outcome | Not aging-specific |
| （99） | Adipocyte lipotoxicity on osteoblasts | Cell (human co-culture) | Normal human osteoblasts co-cultured with differentiating pre-adipocytes, with and without fatty-acid synthase inhibition | Mineralization, alkaline phosphatase, osterix, osteocalcin, Runx2 | Two-chamber co-culture over three weeks; no in vivo or aged-tissue validation | Not aging-specific (human co-culture) |
| （101） | Free fatty acid, ROS and p53 mitochondrial apoptosis | Human observational and animal disease model | Patients with type 2 diabetes; db/db and high-fat-fed mice treated with pathway inhibitors | BMD, bone breaking strength, osteoblastogenesis, mitochondrial apoptosis | Diabetes and high-fat feeding rather than aging; the human component is correlational | Disease-associated (type 2 diabetes); aging-adjacent (high-fat feeding) |
| （90） | Marrow adipocyte RANKL | Cell (primary human) | Primary human bone-marrow adipocytes co-cultured with human osteoclast precursors, with dexamethasone | RANKL expression and RANKL to osteoprotegerin ratio, osteoclast differentiation and resorption | RANKL induction required glucocorticoid exposure and the effect was contact-dependent; no in vivo data | Not aging-specific (glucocorticoid-driven human cells) |
| （86） | LepR-positive stromal cells as the main source of adult bone | Animal genetic (fate mapping) | LepR-Cre fate mapping in adult mouse marrow; transplantation; regeneration after irradiation or fracture | Bone, cartilage and adipocyte fate; regenerated bone | Fate mapping in adults without an aging comparison; the regeneration readouts use injury stimuli | Not aging-specific (adult fate mapping); aging-adjacent (irradiation, fracture) |
| （87） | LepR promotes adipogenesis and reduces osteogenesis | Animal genetic with dietary challenge | Prx1-Cre;Lepr-flox mice with deletion restricted to limb marrow stroma; high-fat diet; marrow stromal cells | Osteogenesis, adipogenesis, fracture healing | Conditional deletion limited to limb stroma; the challenge is dietary rather than age-related | Not aging-specific (conditional genetics); aging-adjacent (high-fat diet) |
| （79） | Marrow adipose volume with age and osteoporosis | Human observational | Iliac-crest biopsies from 53 healthy individuals aged 30 to 100 years and 26 patients with osteoporosis; sex not reported | Marrow adipose, hematopoietic and trabecular bone volume fractions | Cross-sectional; single skeletal site; sex composition not reported; osteoporosis group not age-matched | Natural aging (human) |
| （103） | Vertebral marrow fat, perfusion and BMD | Human observational | 82 older men, mean age 73 years, range 67 to 101 | Vertebral marrow fat content, marrow perfusion, DXA BMD category | Men only; cross-sectional; single vertebral region; perfusion requires contrast | Natural aging (human, men) |
| （104） | Proton-density fat fraction and R2* in osteoporosis | Human observational | 51 patients, 28 female, mean age 69.7 years; 173 vertebral bodies analyzed | Vertebral proton-density fat fraction, R2*, DXA-based category | Cross-sectional; discrimination was moderate (area under the curve 0.66 for fat fraction); analysis at vertebra rather than patient level | Natural aging (human, mixed) |
| （107） | Fat fraction and vertebral fracture | Human observational (case-control) | 52 patients, 63 to 70 per cent women, mean ages 66 and 69 years; fracture cases frequency-matched for age and sex | Osteoporotic vertebral fracture; trabecular BMD by quantitative computed tomography | Small case-control including seven high-energy traumatic fractures; cannot establish temporal order | Natural aging (human, mixed) |
| （110） | Serum leptin and BMD | Human observational (meta-analysis) | 45 studies in healthy populations, literature searched to July 2014 | BMD and bone mineral content | Pooled correlations across heterogeneous studies; body composition is a strong confounder; literature ends in 2014 | Natural aging (human, mixed ages) |
| （112） | Circulating adiponectin, leptin and vaspin with BMD | Human observational | 386 community-dwelling postmenopausal women, mean age 61 years | Lumbar spine, femoral neck and total hip BMD | Cross-sectional; the inverse adiponectin association was confined to women with osteoporosis at one site | Natural aging (human, postmenopausal) |
| （114） | Adiponectin and fracture risk over time | Human observational (prospective) | 989 older men, MrOS Sweden, mean follow-up 5.3 years, 124 with fracture | Incident osteoporotic fracture | Men only; the gradient of risk attenuates with time since baseline, limiting long-term prediction | Natural aging (human, men) |
| （17） | Senescent cells in the bone microenvironment | Animal (aged versus young) | Young (6 months) and old (24 months) mice of both sexes; enriched bone and marrow populations isolated without culture | p16Ink4a expression, senescence-associated distension of satellites, telomere dysfunction foci, SASP markers by cell type | Marker-based cross-sectional comparison without intervention; no human tissue | Natural aging |
| （18） | Targeting senescent cells in aged bone | Animal genetic and pharmacological | Aged mice (20 to 22 months) with established bone loss; INK-ATTAC transgene, senolytics, or JAK inhibitor for 2 to 4 months | Bone mass, strength and microarchitecture; resorption and formation indices; osteoblast and osteoclast assays in vitro | Genetic clearance is not a selective blockade of the secretome and JAK inhibition has additional actions, so causality is supported but not isolated | Natural aging |
| （135） | T-cell TNF-alpha in estrogen deficiency | Animal genetic with surgical model | Ovariectomized mice, including T-cell-deficient animals | Bone loss, bone resorption, macrophage colony-stimulating factor and RANKL-driven osteoclastogenesis | Acute surgical estrogen withdrawal; T-cell-deficient mice carry broader immune defects | Aging-adjacent (ovariectomy) |
| （137） | Marrow TNF-producing T cells | Animal genetic with surgical model | Ovariectomized wild-type, TNF-null, nude, and TNF-receptor-null mice; adoptive T-cell transfer | Bone loss; number of TNF-producing marrow T cells | Same acute ovariectomy model; adoptive transfer is a reconstitution rather than a physiological state | Aging-adjacent (ovariectomy) |
| （138） | Class II transactivator, T-cell lifespan and estrogen loss | Animal with surgical model | Ovariectomized mice; blockade of antigen presentation; silencing of interferon-gamma receptor signaling | Bone loss; T-cell proliferation and lifespan; antigen presentation | Same acute model; the mechanism is inferred from blockade experiments | Aging-adjacent (ovariectomy) |
| （154） | TNF-alpha blunts osteogenic effects of muscle vesicles | Cell | TNF-alpha-treated C2C12 cells; mouse marrow, RAW264.7 and ST-2 cells | Osteoclast formation, osteoblastic differentiation markers, vesicular miR-196a-5p content | Entirely in vitro; the cytokine is applied to the donor muscle cells rather than to bone tissue | Not aging-specific |
| （155） | TNF-alpha inhibits osteoblast differentiation | Cell | Fetal calvarial precursor cells and the MC3T3-E1-14 clonal line | Multilayered nodule formation, mineralization, osteocalcin secretion | Fetal and immortalized cells; cytokine exposure may exceed tissue concentrations | Not aging-specific |
| （156） | TNF-alpha suppresses RUNX2 | Cell | Fetal calvarial precursor cells and MC3T3-E1 cells; promoter-reporter and mobility-shift assays | RUNX2 messenger RNA, nuclear content and promoter activity | In vitro transcriptional readouts only; no bone outcome measured | Not aging-specific |
| （157） | Dickkopf-1 as a regulator of joint remodeling | Animal disease model and human tissue | Mouse inflammatory arthritis model; human rheumatoid arthritis samples | Bone-destructive versus bone-forming pattern at the joint | Arthritis-driven joint remodeling rather than age-related remodeling of the skeleton | Disease-associated (inflammatory arthritis) |
| （127） | Independence of estrogen deficiency and senescence | Human interventional and animal surgical | 10 elderly postmenopausal women given 3 weeks of estrogen therapy versus 10 untreated; young adult ovariectomized, orchidectomized and INK-ATTAC mice | Senescence biomarkers and SASP in bone biopsies; trabecular and cortical bone after ovariectomy | Very small human groups with a 3-week exposure and no young comparator; the mouse arm is a young-adult acute model, so neither arm measures accumulation with age | Natural aging (elderly human cohort, with estrogen-therapy intervention); aging-adjacent (ovariectomy in mice) |
| （129） | Inflammatory bone proteome across age | Human observational (proteomic) | Bone tissue from children, middle-aged and elderly donors; sex not reported | Differentially expressed proteins; inflammatory and bone-formation pathways | Group-level proteomics without cell-type resolution; drug predictions are computational and only partly validated | Natural aging (human) |
| （136） | Marrow RANKL in early postmenopause | Human observational | 12 premenopausal, 12 early postmenopausal and 12 estrogen-treated postmenopausal women | Surface RANKL per marrow cell by flow cytometry; resorption markers | Small groups; cross-sectional; a marker-level rather than an outcome-level endpoint | Natural aging (human, postmenopausal) |
| （153） | Osteocalcin signaling in myofibers | Animal genetic and pharmacological | Mice, monkeys and humans for the decline in circulating osteocalcin; myofiber-specific receptor deletion; exogenous osteocalcin in young and 15-month-old mice | Exercise capacity, nutrient uptake and catabolism, exercise-induced IL-6 release | The measured outcome is muscle rather than bone, and the human component is a circulating-level observation only | Natural aging (age-related decline; aged mice restored) |

Supplementary Table S1. Study characteristics, limitations and aging context of the reports underlying Table 1 and Table 2. Rows cover every study cited in the edge list of Table 1 and in the human evidence of Table 2, deduplicated, and are ordered by arm: muscle-derived and muscle-associated signals, fat-derived signals, senescence-associated and immune signals, and the reverse bone-to-muscle edge. ‘Study model’ gives the class of experiment (cell, animal genetic, animal pharmacological, mechanical or dietary challenge, human observational, human interventional). ‘Population or system’ gives the species, line, cohort, and, where the source reports them, the sample size, age range and sex composition; these fields are left unstated where the source does not report them. ‘Skeletal outcome measured’ names the endpoint actually assayed, which for some entries is a cellular or marker endpoint rather than bone mass or fracture. ‘Major limitation’ summarizes the principal design-based constraint on interpretation identified during our appraisal of each study. ‘Aging context’ uses the same four categories as Table 1, namely Natural aging, Aging-adjacent, Disease-associated and Not aging-specific, with study-specific detail added; rows carry more than one value where a single report combines designs. Study characteristics were compiled from PubMed records and verified against full texts where necessary. Populations and endpoints were extracted from the published reports, whereas the ‘Major limitation’ column summarizes the principal design-based constraint identified during our appraisal. This review is a narrative synthesis and no exhaustive search string was registered, as stated in Section 1.1; this table therefore documents the characteristics of the evidence cited rather than the yield of a systematic search. Numbers in the first column refer to the numbered reference list of the main text.
